# Human perceptual and metacognitive decision-making rely on distinct brain networks

**DOI:** 10.1371/journal.pbio.3001750

**Published:** 2022-08-09

**Authors:** Paolo Di Luzio, Luca Tarasi, Juha Silvanto, Alessio Avenanti, Vincenzo Romei

**Affiliations:** 1 Center for Studies and Research in Cognitive Neuroscience, University of Bologna, Cesena, Italy; 2 School of Psychology, Faculty of Health and Medical Sciences, University of Surrey, Guildford, United Kingdom; 3 Centro de Investigación en Neuropsicología y Neurociencias Cognitivas, Universidad Católica del Maule, Talca, Chile; 4 IRCCS Fondazione Santa Lucia, Rome, Italy; McGill University, CANADA

## Abstract

Perceptual decisions depend on the ability to exploit available sensory information in order to select the most adaptive option from a set of alternatives. Such decisions depend on the perceptual sensitivity of the organism, which is generally accompanied by a corresponding level of certainty about the choice made. Here, by use of corticocortical paired associative transcranial magnetic stimulation protocol (ccPAS) aimed at inducing plastic changes, we shaped perceptual sensitivity and metacognitive ability in a motion discrimination task depending on the targeted network, demonstrating their functional dissociation. Neurostimulation aimed at boosting V5/MT+-to-V1/V2 back-projections enhanced motion sensitivity without impacting metacognition, whereas boosting IPS/LIP-to-V1/V2 back-projections increased metacognitive efficiency without impacting motion sensitivity. This double-dissociation provides causal evidence of distinct networks for perceptual sensitivity and metacognitive ability in humans.

## Abstract 2

Transcranial magnetic stimulation targeting cortico-cortical connections reveals a functional dissociation between temporo-visual and parieto-visual re-entrant pathways in humans, controlling perceptual sensitivity and metacognitive abilities respectively, during a visual motion perception task.

## Introduction

The ability to exploit available sensory information in order to select the most adaptive option from a set of alternatives represents a fundamental decisional skill. Once a perceptual judgment about a stimulus is made, the resulting subjective belief that the perceptual decision is correct is referred to as confidence. Evaluation of confidence can be intended as a metacognitive process, since it represents a postdecisional outcome regarding the accuracy of first-order choice [1,2]. These components of perceptual decision-making may appear intrinsically intertwined, and yet, recent behavioral and neural findings hint at a possible functional dissociation between performance accuracy and confidence [3–5].

From a behavioral perspective, accuracy and confidence in perceptual decision have been frequently dissociated in nonhuman primates [6] and humans [7–10]. There is empirical evidence of simple dissociations between perceptual sensitivity and metacognitive processes in the form of selective perturbations of confidence without alterations of discriminative performance [11,12]; in fact, the existence of a metacognitive noise has been proposed to describe sources that selectively influence confidence generation, such as previous trials bias [13], arousal [14], or fatigue [15]. Behavioral evidence is further supported by the existence of specific neural correlates, which suggest distinct computations underlying sensory decisions and metacognitive abilities [16,17]. However, it is still unclear whether it is possible to actively induce a targeted modulation of perceptual sensitivity and metacognition by intervening on the efficiency of the cortical networks underlying these components of perceptual decisions.

Perceptual decision-making studies have classically focused on visual motion processes [18]. These studies have suggested that neurons in the middle temporal area (V5/MT+), which are tuned to the direction of motion stimuli, are essential for perceptual sensitivity, ultimately leading to accurate motion discrimination [19].

Moreover, electrical microstimulation in animals [20] has confirmed the causal role of V5/MT+ in representing sensory evidence, showing that enhanced perceptual discrimination is possibly driven by signal amplification mechanisms, which may in turn influence confidence generation [21]. Conversely, other animal studies pointed to the fundamental role of the lateral intraparietal cortex (LIP) in shaping the decision process per se [22]. Decision certainty modulations have been found during LIP stimulation in monkeys [23], in line with the notion that this area and the corresponding intraparietal sulcus (IPS) in humans [24,25] are implicated in choice formation [26,27].

Visual awareness for global coherent motion (i.e., evidence of movement) has been shown to require the recruitment of feedback pathways from V5/MT+-to-V1/V2 [28,29]. Such connections can be transiently strengthened by means of a novel transcranial magnetic stimulation (TMS) protocol, based on the Hebbian principle, namely the corticocortical paired associative stimulation (ccPAS) [30–34]. This noninvasive stimulation implies a repetitive activation of interconnected cortical sites at specific interstimulus intervals, which are based on the timing of physiological communication between targeted areas, so to mimic patterns of neuronal stimulation shown to induce spike timing–dependent plasticity (STDP)—a form of synaptic plasticity meeting the Hebbian principle that synapses are potentiated if the presynaptic neuron fires immediately before the postsynaptic neuron in a coherent and repeated manner [35–37]. ccPAS has proved capable of modifying neurophysiological responses [38–41] and recently opened the possibility of testing its behavioral consequences [42,43], such as leading to enhanced perceptual discrimination of coherent motion when targeting V5/MT+-to-V1/V2 back-projections [44,45].

Yet, the relevance of IPS/LIP in specific aspects of decision confidence [23,46], in association with evidence of back-projections from parietal to early visual areas [47–49] and their possible role in visual awareness [50,51], raises the question about the functional role of these latter parieto-occipital connections in perceptual decision-making. Indeed, a fundamental and yet unanswered question is whether and how do IPS/LIP-to-V1/V2 back-projections functionally contribute to decision-making process, including its confidence.

Here, we specifically sought to dissociate the functional role of V5/MT+-to-V1/V2 and IPS/LIP-to-V1/V2 networks in motion perception decisions by means of ccPAS. In line with previous findings [44,45], ccPAS aimed at strengthening V5/MT+-to-V1/V2 back-projections is expected to enhance coherent motion perception. Crucially, assuming IPS/LIP major involvement in decision processes [22], decision certainty modulations [23] and choice formation [26,27]; information-based [32] IPS/LIP-to-V1/V2 ccPAS is expected to drive shifts in choice-related metacognitive awareness, without impacting perceptual sensitivity per se. Conversely, parieto-occipital stimulation lacking STDP specificity should not lead to behavioral modifications.

## Results and discussion

**Fig 1:**
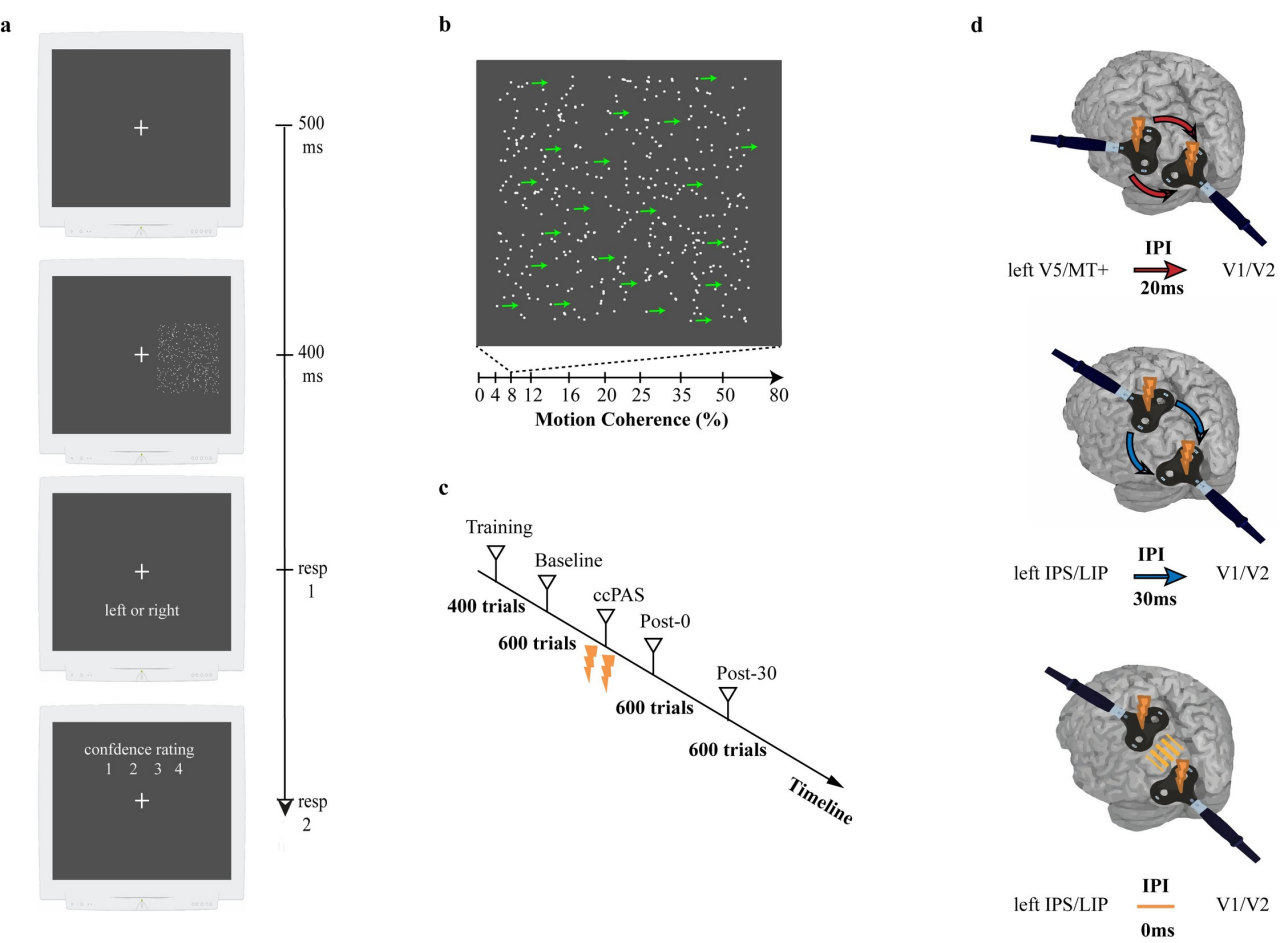
Experimental paradigm. **(a)** Task sequence. Each trial started with a fixation cross-displayed for 500 ms followed by a dot motion stimulus displayed for 400 ms, presented on the right side of the cross. Participants were requested to press a response key immediately after the offset of the stimulus, by selecting the coherent motion perceived (e.g., leftward or rightward) and subsequently to report their confidence by pressing the respective numeric keys (e.g., 1,2,3,4). No time out was present for both responses. (**b)** Stimuli. The motion coherence of the stimulus varied across trials (ranging from 0% up to 80%, across 10 levels); here a schematic representation of a stimulus with 8% of dots moving rightward. (**c)** Experiment timeline. For each participant, the experiment began with a training session of 2 blocks, performed to allow the participant to reach a stable performance level before the actual experiment. This preliminary phase was followed by a BSL session. After the BSL measurement, participants were randomly assigned to one of 2 ccPAS conditions. Participants had to perform the same task immediately (T0), and 30 (T30) minutes following the ccPAS protocol. One session consisted of three blocks of 200 trials each. (**d)** ccPAS protocols. The stimulation lasted 15 minutes and consisted of 90 paired pulses at fixed intensity (60% of TMS max output). The parameters and cortical target varied relative to the pathway involved. In particular, the IPI between stimulated areas was set to 20 ms for Exp_*V5-V1*_, 30 ms for Exp_*IPS-V1*_, and 0 ms for Ctrl_*IPS-V1*_. BSL, baseline; ccPAS, corticocortical paired associative stimulation; IPI, interpulse interval; TMS, transcranial magnetic stimulation.

Fifty-one participants were requested to determine the horizontal direction of a dots pattern in a discrimination task (Fig 1B), in which trials varied across a percentage gradient of motion coherence (i.e., 10 levels from low to high coherence, see Methods), and subsequently rated the confidence on their response (Fig 1A). Sensory discrimination and response certainty were assessed in a between-subjects design before and after the administration of the ccPAS protocol (Fig 1D) over 2 key networks involved in perceptual decision, namely, V5/MT+-to-V1/V2 pathway (i.e., Exp_V5-V1_) and IPS/LIP-to-V1/V2 pathway (i.e., Exp_IPS-V1_), with the relative control condition (i.e., Ctlr _IPS-V1_). Participants underwent the ccPAS procedure following a baseline (BSL) assessment, after which they repeated the initial measure in 2 testing sessions immediately after (T0) and 30 minutes after ccPAS (T30) (see Methods) (Fig 1C).

**Fig 2:**
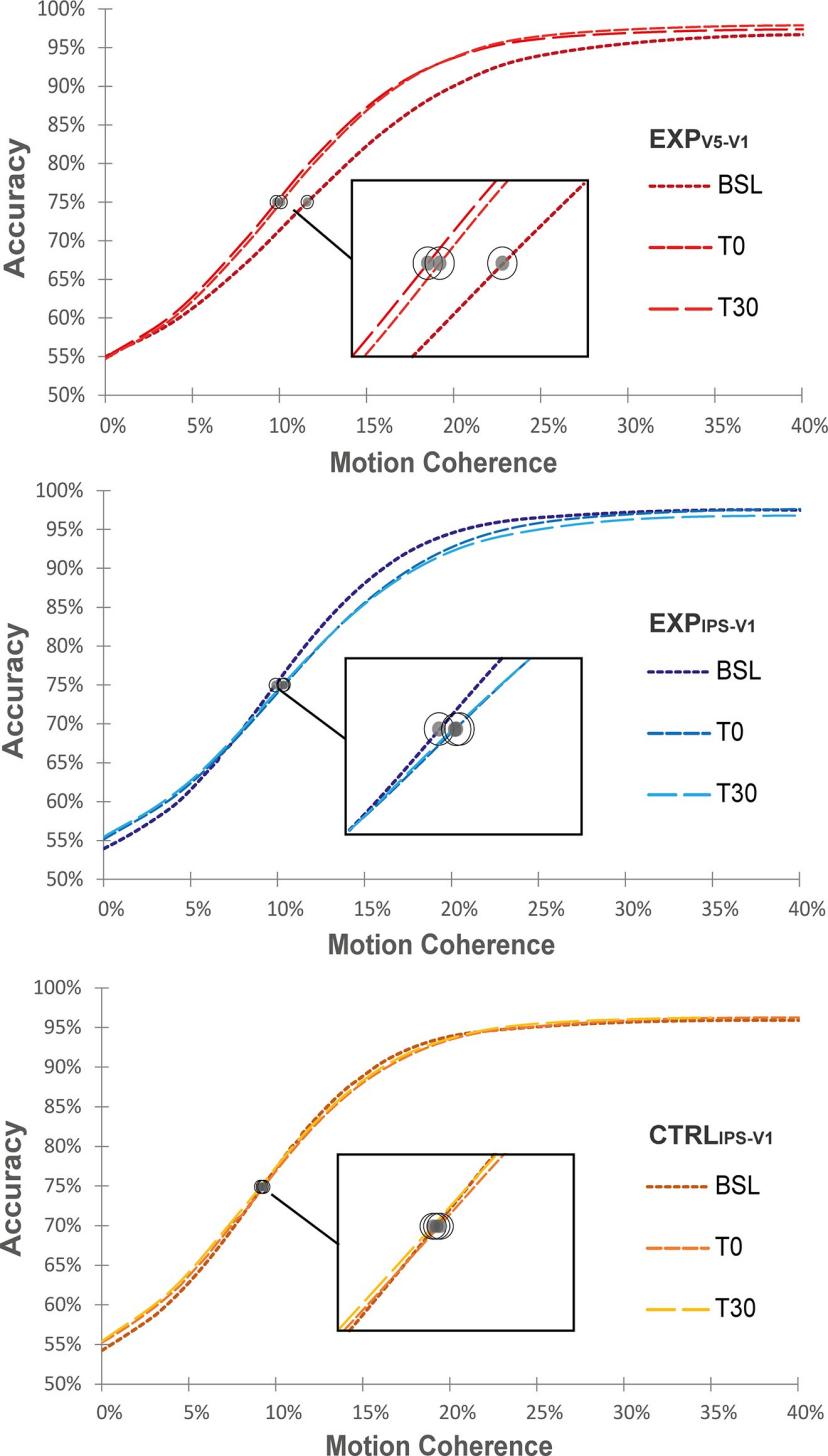
Psychometric curves. Fitted data modeled on the logistic function to obtain the perceptual thresholds of motion discrimination. Group performance are separately plotted depending on the type of stimulation (top graph, in red **Exp**_**V5-V1**_; mid graph, in blue **Exp**_**IPS-V1**_; bottom graph, in yellow **Ctrl**_**IPS-V1**_) and as a function of the session. Gray dots depict the perceptual threshold coincident with the percentage of coherent motion where the logistic function had a value of 75% of correct responses. Perceptual thresholds shifts on the abscissa represent lower (right-shift) or higher (left-shift) motion sensitivity. Data underlying this figure can be found in OSF: https://osf.io/x7d2e/?view_only=ac2ff19b1ab6415cb471895854fb5a35.

**Fig 3:**
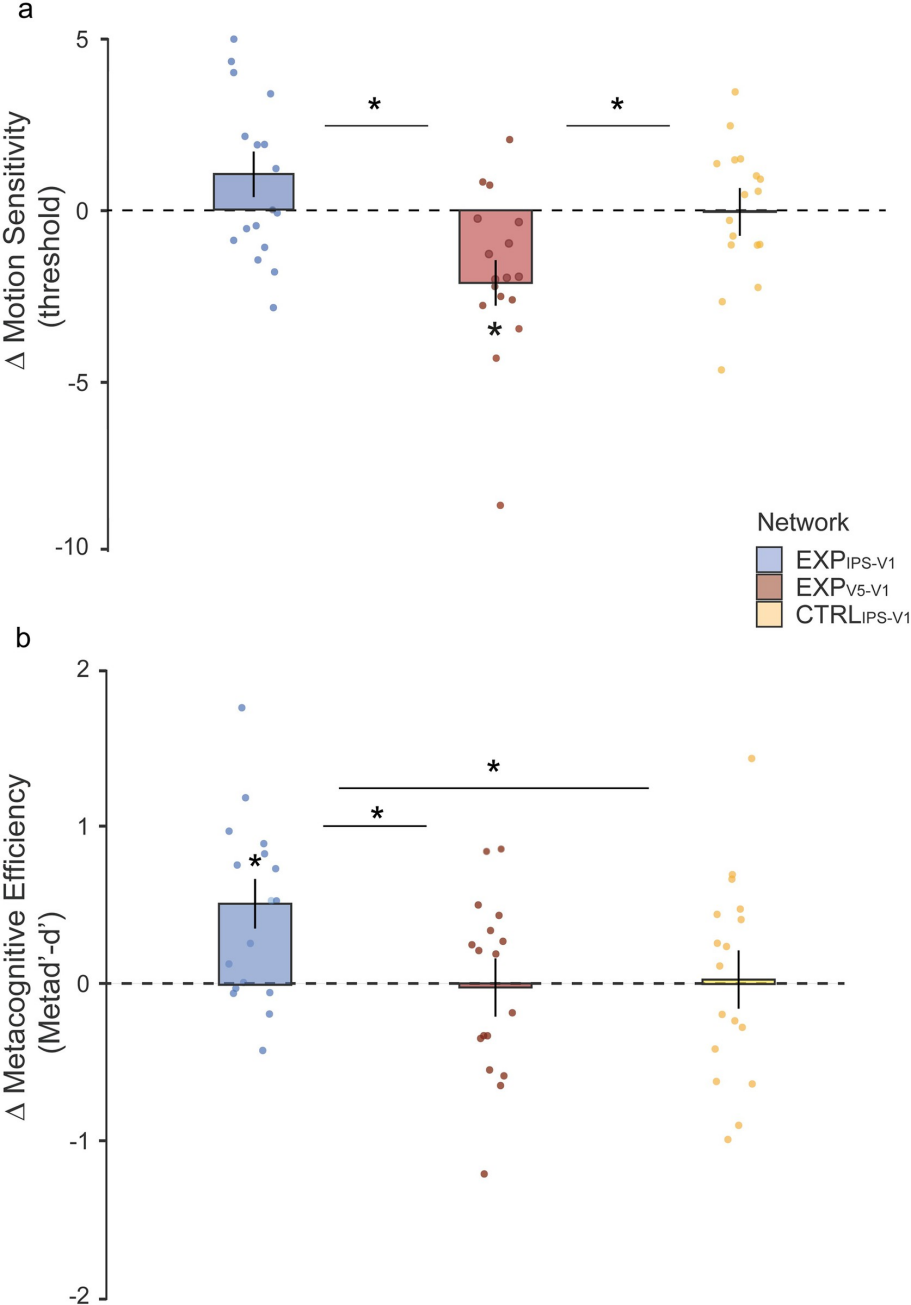
ccPAS effect on decision-making. **(a)** Motion threshold following stimulation. Filled bars represent the mean change Δ in sensitivity threshold (e.g., differences between Post ccPAS and BSL), and error bars represent the SEM. Individual data points are plotted by scattered dots. Asterisks point out significance (*p < .05) for Exp_V5-V1_ mean and between group means. (**b)** Metacognitive efficiency following stimulation. Filled bars represent the mean change Δ in metacognition with error bars representing SEM. Individual performances are plotted by scattered data points. Asterisks point out significance (*p < .05) for Exp_IPS-V1_ mean and between group means. Data underlying this figure can be found in OSF: https://osf.io/x7d2e/?view_only=ac2ff19b1ab6415cb471895854fb5a35. BSL, baseline; ccPAS, corticocortical paired associative transcranial magnetic stimulation protocol.

Analyses performed on baseline-corrected motion sensitivity threshold (see Fig 2 for group psychometric curves) across the Exp_V5-V1_, Exp_IPS-V1_, and Ctlr_IPS-V1_ (i.e., Targeted Network factor) ccPAS conditions depending on the time from stimulation (i.e., Time factor: T0, T30) revealed a significant impact of ccPAS condition (Main effect of Targeted Network: F_2,48_ = 6.51; *p* = .003; np^2^ = .21) irrespective of the session (Targeted Network*Time: F_2,48_ = .27; *p* = .76; np^2^ = .01), showing larger improvements following Exp_V5-V1_ ccPAS relative to Exp_IPS-V1_ ccPAS (*p* = .001) and relative to Ctlr_IPS-V1_ (*p* = .02) (Fig 3A). Relative to baseline, motion discrimination abilities significantly improved following ccPAS targeting of V5/MT+-to-V1/V2 back-projections, as reflected by a reduction of sensitivity threshold (Exp_V5-V1,_ Avg T0+T30: Mean = −1.85; SEM = .58; *p* = .02; Cohen’s d = −.77), as expected [44,45]. Crucially, such effect was selective for Exp_V5-V1_ ccPAS as no significant modulation in perceptual accuracy could be observed following ccPAS targeting the IPS/LIP-to-V1/V2 reentrant pathway (Exp_IPS-V1,_ Avg T0+T30: Mean = .88; SEM = .57; *p* = .28; Cohen’s d = .38) or the IPS/LIP-V1/V2 network (Ctrl_IPS-V1,_ Avg T0+T30: Mean = −.01; SEM = .48; *p* = .97; Cohen’s d = −.008), thus confirming the causal role of the V5/MT+-to-V1/V2 pathway in global motion sensitivity and showing its anatomical specificity. These results were corroborated by a Bayesian analysis, revealing that the model including the ccPAS condition (i.e., the Targeted Network factor) better predicts performance (BF_inclusion_ = 14.99) relative to models excluding it, and data about motion sensitivity of Exp_*V5-V1*_ consistently support the hypothesis of the improvement (BF_10_ = 8.55) relative to Exp_IPS-V1_ (BF_10_ = .69) and Ctrl_IPS-V1_ (BF_10_ = .25). Additional analysis on raw behavioral measures have also been reported on Supporting information (S1 File) along with additional plots (S1–S3 and S4 Figs) further detailing the nature of these effects.

The impact of ccPAS over V5/MT+-to-V1/V2 and IPS/LIP-to-V1/V2 networks was then tested on metacognitive efficiency, indexed by the matching between confidence attribution and perceptual sensitivity (i.e., difference between meta-d’ and d’; see Methods) at participant’s threshold levels. Modulation of this metacognitive index was again dependent on the ccPAS condition (Main effect of Targeted Network: F_2,48_ = 3.29; *p* = .046; np^2^ = .12), with larger improvements in metacognition abilities in the Exp_*IPS-V1*_ relative to the Exp_*V5-V1*_ (*p* = .03) and relative to Ctlr_IPS-V1_ (*p* = .04) conditions, independently of session (Targeted Network*Time: F_2,48_ = .03; *p* = .97; np^2^ = .001). Participants showed increased metacognition following ccPAS targeting the IPS/LIP-to-V1/V2 pathway (Exp_IPS-V1,_ Avg T0+T30: Mean = .46; SEM = .14; *p* = .01; Cohen’s d = .79) relative to baseline. No modulation in metacognitive efficiency was observed following ccPAS targeting the V5/MT+-to-V1/V2 network (Exp_V5-V1,_ Avg T0+T30: Mean = −.02; SEM = .14; *p* = .89; Cohen’s d = −.03) or nonspecific stimulation of the parieto-occipital network (AVG T0+T30: Mean = .02; SEM = .16; *p* = 1; Cohen’s d = .04) (Fig 3B). Consistently, Bayesian ANOVA confirmed that metacognitive data were adequately explained (BF_inclusion_ = 1.79) by a model including the ccPAS condition (i.e., Targeted Network), supporting a metacognition improvement in Exp_IPS-V1_ (BF_10_ = 11.16), but not in Exp_*V5-V1*_ (BF_10_ = .25) or Ctrl_IPS-V1_ (BF_10_ = .25). Supplementary analysis on metacognition and raw confidence data, with related plots, have been reported separately (S5 and S6 Figs), further detailing the nature of these effects. Briefly, additional analyses on raw data highlight a different impact of the stimulation group on confidence rating for correct and error responses. Specifically, we found that following IPS/LIP-to-V1/V2 ccPAS, participants confidence increased exclusively for correct trials (t = 2.87; *p* = .04) with no alteration for error trials (t = 1.65; *p* = .12). In contrast, following V5/MT+-to-V1/V2 ccPAS, participants showed a general increase in confidence, independently of whether their responses were correct (t = 3.62; *p* = .01) or incorrect (t = 3.29; *p* = .02), speaking in favor of a specific role of IPS-V1 back-projections in sensory processing readout (S6 Fig).

Here, we showed that distinct visual networks can be functionally dissociated when investigating metacognitive functions, complementarily to perceptual discrimination performance. These effects cannot be alternatively explained by simple time passing—which in principle might have made participants more efficient at rating their confidence over time—or any unspecific effect of TMS. Nonspecific stimulation of the parieto-occipital stream (Ctrl_IPS-V1_) showed no modulatory effects in terms of motion sensitivity or metacognitive functions. Moreover, prior work using the same motion task has shown no change in perceptual sensitivity following sham or ineffective stimulation of the V5/MT+-to-V1/V2 network [44,45].

Our findings provide causal evidence of a double dissociation of functional networks orchestrating perceptual decision-making in humans, namely, V5/MT+-to-V1/V2, accounting for visual motion discrimination sensitivity, and IPS/LIP-to-V1/V2, accounting for accurate confidence judgments. In line with current opinions [3,52] that sensitivity and confidence could be served by partially distinct processes, we reported for the first time that the TMS protocol aimed at enhancing IPS/LIP-to-V1/V2 pathway selectively affects metacognitive capacity in a functional way, with participants becoming effectively more accurate in estimating the quality of their choices (i.e., more accurate in their confidence ratings).

Crucially, we found evidence of a functional segregation of targeted networks. The enhanced metacognitive capacity did not lead to simultaneous increase of motion sensitivity, being this a function subserved by another network. Indeed, ccPAS over V5/MT+-to-V1/V2 back-projections was critical in increasing motion sensitivity and accuracy, as expected [44,45].

These findings challenge the view that higher perceptual accuracy, as the one induced by ccPAS over V5/MT+-to-V1/V2 back-projections, may produce a modulation of metacognitive functions due to a finer discrimination of the stimuli, as assumed by a model where perceptual decision and confidence are based on a common underlying neural representation [21,53]. This interpretation would not explain why ccPAS over V5/MT+-to-V1/V2 back-projections does not lead to enhanced metacognitive efficiency. Instead, it supports the notion that confidence generation and perceptual sensitivity are supported by relatively independent mechanisms [54–56]. In detail, the sensory representation necessary for the perceptual readout would not be the absolute source for metacognitive estimation, being the latter the result of an accumulation process, which integrates further information after, or even while the perceptual choice is made [16,57]. This mechanism would admit conditions in which alterations of sensory representation produce divergences between perceptual and metacognitive outcomes [7,58], and seems to be suggested by secondary evidence showing a bias in confidence generation following V5/MT+-to-V1/V2 stimulation (see Text B in S1 File), leading the subjects to nonspecific overestimation of certainty, without altering their metacognitive efficiency (Text C in S1 File).

On the other hand, neurostimulation aimed at enhancing the IPS/LIP-to-V1/V2 back- connectivity improved metacognitive ability without impacting motion sensitivity. This effect may be possibly sustained by an optimized performance to confidence degree mapping (Text B and C in S1 File). Consistently to what had already been demonstrated for the V5/MT+-to-V1/V2 network [45,46], here, the effect was conditional to the causal order of the pulses, since no outcome could be observed when controlling for timing (Ctrl_IPS-V1_). This result, taken together with previous findings reporting the selective manipulations of confidence without affecting accuracy [7,16,59,60], is in line with the proposal that considers metacognition as a distinct functional process [2,59,61]. This implies a system where the actual computations that underlie these 2 processes may be sustained by dissociable, perhaps both in time and spatial scale, neural circuits [57]. Given this, it is crucial to address what could be a plausible neural mechanism for the overall pattern of results. Assuming distinct elaboration levels for perceptual decision and confidence, the activity emerging from V5/MT+-to-V1/V2 network appears fundamental for sensory discrimination. This reentrant pathway subserves an adaptive mechanism that adjusts local circuitry of V1 to highlight output of cells representing salient information and suppress others irrelevant information [62], thus optimizing representation of the predicted trajectory in MT+/V5 to overcome direction uncertainty, and improving the final readout. As previously mentioned, the sensory evidence is then plausibly involved in the transformations needed for metacognitive estimation; however, this step incorporates other sources, identified as metacognitive noise [63], leading to a distinct representation. We propose that the improved metacognitive performance following IPS/LIP-to-V1/V2 stimulation may be a consequence of decreasing noise in confidence generation, as already suggested in other studies with neuromodulation on metacognition [55,64]. In this case, reduction of metacognitive noise could derive from an efficient gating of early visual areas (V1-V2) from parietal area (IPS/LIP) by back-projections, possibly orchestrating neuronal firing with a fine balance between excitation and inhibition [65]. The suggested process would reduce the uncertainty estimates in the circuit for metacognitive computation by stabilizing the neural activity in early visual cortex. This function is not at odds with the alpha frequency-specific activity attributed to feedback influence [66,67], mediating inhibitory and disinhibitory effects on visual areas [68–71]. Consistently with the hypothesis of distinct functional computations for accuracy and confidence, the abovementioned mechanism would selectively shape the representation of confidence without affecting the first-order representation used for sensory discrimination, possibly tuning specific oscillatory parameters.

Interestingly, a recent preprint supports the critical involvement of alpha oscillations in feedback activity by showing an association between boosting V5/MT+-to-V1/V2 connectivity and alpha modulation [72]. These findings are very intriguing for the current report in the light of recent evidence for the role of different parameters of alpha oscillations in generating perceptual sensitivity versus confidence and metacognitive functions [5]. Importantly, metacognitive abilities have been shown to correlate with modulation in alpha amplitude only following stimulus presentation, in line with the idea of poststimulus choice metacognitive readout.

By taking into account the evidence that early visual areas and higher-order regions constitute a recurrent feedback system [73], we could also hypothesize the existence of a hierarchical Bayesian architecture in which looping iterations tend to perform near-optimal computations [74,75], combining and updating predicted and observed input to reduce uncertainty. Empirically, it has been reported that in recurrent circuit models of decision-making, reentrant pathways continuously propagate the evolving decision variable from upstream regions to sensory regions [76,77], and the state of the early visual cortex is shaped by an adaptive, stabilizing feedback of the evolving decision variable [78,79]. In a related line of reasoning, the parietal node may serve a higher-order supervisory function feeding back lower-level areas and thus integrating recursive information across the hierarchy. For example, comparing the expected sensory signal as computed in V5/MT+ (motion direction) with the effective signal update recorded in early visual areas (actual stimulus position) may provide a near-optimal mechanism modulating confidence levels depending on the match between expected and actual sensory signal in V1/V2. Small differences between expected and actual sensory signal computation prompt maximum confidence and vice versa. Recursive cycles between IPS/LIP and V1/V2 would then promote the metacognitive awareness associated with the task.

It should be noted that a previous attempt of active manipulation in posterior parietal cortices by means of TMS failed to trace any effect on metacognitive functionality [80]. This outcome was presumably due to a different cortical site location and a distinct stimulation paradigm employed relative to ours. Nevertheless, the potential of TMS at dissociating choice component of accuracy and confidence has been proven extensively in other works, mainly involving the causal manipulation of the prefrontal cortex [11,81] and early visual areas [4,82]. Here, we provide the first evidence of a causal involvement of the functional pathway from parietal to early visual areas in metacognitive processes. In light of these results, we also consider reasonable that an increased functionality of the parieto-occipital stream at integrating perceptual information might favor the metacognitive readout mechanism that occurs from the communication between frontal (e.g., BA10) and parietal (e.g. LIP/IPS) regions [83]. This seems a plausible explanation given the existence of a series of recursive chains that are diffused along the frontoparietal axis [84,85]; moreover, it appears in line the notion of a long-range distributed network involved in metacognition [57,86]. Yet, regardless of the extension of the specific brain network involved, our findings support a critical role for IPS-to-V1/V2, rather than V5/MT+-to-V1/V2 pathways in visual metacognition.

In conclusion, we functionally dissociate the role of V5/MT+-to-V1/V2 and IPS/LIP-to-V1/V2 back-projections in perceptual decision processes. Our findings provide evidence supporting a selective modulation of perceptual sensitivity through signal amplification by V5/MT+-to-V1/V2 back-projections and metacognitive efficiency through uncertainty supervision by IPS/LIP-to-V1/V2 back-projections speaking in favor of distinct but integrated systems subserving near-optimal perceptual decision processes in humans.

## Materials and methods

### Experimental design

#### Participants

Fifty-one healthy individuals were recruited for the study. Participants were divided into 3 groups of 17 participants each and submitted to the V5/MT+-to-V1/V2 ccPAS condition (Exp_*V5-V1*_, 8 males); the IPS/LIP-to-V1/V2 ccPAS condition (Exp_*IPS-V1*_, 9 males); or the IPS/LIP-V1/V2 control condition (Ctrl_*IPS-V1*_, 7 males). Participants’ ages varied between 20 and 28 years. Sample size was determined by power analysis based on related works [44,45] (effect size f = .25, alpha = .05, and 90% power). All studies were approved by the University of Bologna Research Ethics Committee. All participants had normal or corrected-to-normal vision. Informed consent was obtained from all participants before the experiment. Participants were all volunteers and received no payment or compensation. All investigators were blinded to any group, participant, or sequence allocation during data collection and analyses.

#### Visual stimuli

Stimuli were presented on a gray background and consisted of 400 white dots (0.21° visual angle) moving within a square region subtending 12.8 × 12.8 degrees of visual angle, which appears on the right side of a white fixation cross (0.72 × 0.72° visual angle) located in the center of the screen on a gray background. In each trial, dots moved with a different level of motion coherence (0%, 4%, 8%, 12%, 16%, 20%, 25%, 35%, 50%, or 80%) leftward or rightward. Motion coherence was expressed as the percentage of dots that were moving in the signal direction. In the 0% coherence trials, all the dots moved randomly; in the 80% coherence trials, 320 dots (80%) moved coherently toward leftward or rightward, while the remaining 80 dots (20%) were each given a randomly selected direction of motion. Each dot moved at a speed of 4.5°/second. The stimuli were presented in an 18-inch screen with a resolution of 1,280 × 1,024 pixels and a refresh rate of 85 Hz. In all tests, stimulus presentation was implemented by MATLAB (R2016b, MathWorks) using the Psychophysics Toolbox [87].

#### Coherent motion direction discrimination paradigm

The task was a random dot motion discrimination task. Each trial began with a fixation cross appearing in the middle of the screen for 500 ms, followed by the stimulus, the duration of which was 400 ms. After each trial, participants were asked to respond by pressing the left arrow or the right arrow key to indicate the perceived global direction of motion. After collecting this response, participants were asked to select the confidence level associated with the direction of motion decision using a discrete scale (1: totally uncertain, 2: uncertain, 3: quite certain, 4: totally certain). Participants had to press keyboard button corresponding to their confidence judgment. A task block consisted of 200 trials: 10 trials × 2 directions (leftward/rightward coherent direction of motion) × 10 coherence levels. Each session consisted of 3 blocks, for a total of 600 trials and it lasted approximately 18 minutes.

#### General procedure

The experiment was a between-subject design carried out in separate sessions. Participants were randomly assigned to 3 different groups according to the TMS protocol they would undergo. After having familiarized themselves with the task and achieving a stable performance on the motion task in a training session, participants performed their BSL session before undergoing their assigned TMS protocol. Participants performed the motion direction discrimination task again, immediately (T0) and 30 (T30) minutes after the stimulation. In accordance with previous studies, we planned specific poststimulation testing sessions in order to monitor the evolution of neural plasticity effects [33], as well as considering that ccPAS aftereffect are usually circumscribed within 60 minutes from stimulation with peaks at around 30 minutes [88–90].

### ccPAS protocol

ccPAS was delivered by means of two 50-mm figure-of-eight coils, connected to a Magstim dual pulse monophasic stimulator (Magstim Company, Whitland). A total of 90 pairs of stimuli were continuously delivered at a rate of 0.1 Hz for approximately 15 minutes, each pair of stimuli consisted of 2 monophasic transcranial magnetic pulses [88]. The pulses were triggered remotely using a Matlab interface that controlled both stimulators. In every condition, intensity of TMS was set at 60% of the maximum stimulator output [28,91,92]. A neuronavigation software (SofTaxic, E.M.S., Bologna, Italy) combined with a 3D optical digitizer (Polaris Vicra, NDI, Waterloo, Canada) was used to control the consistency of EEG scalp position with the mean MNI coordinates of the involved cortical site. The SofTaxic software estimated the volume of magnetic resonance images of the subject’s head by means of a warping procedure, on the basis of the subject’s skull landmarks (nasion, inion, and 2 preauricular points) and a set of 65 points providing a uniform representation of the scalp. The ccPAS protocol was manipulated in 3 different groups of participants. In one group we stimulated the V5/MT+-V1/V2 network and the other two groups we stimulated the IPS-V1/V2 network, as specified below.

#### Experimental ccPAS condition V5/MT+-to-V1/V2 (Exp_V5-V1_)

Left V5/MT+ and central V1/V2 were stimulated using established procedures [44,45]. To target left V5/MT+, the coil was centered 3 cm dorsal and 5 cm lateral to the inion (S7 Fig), corresponding to the average functionally localized scalp position where perception of moving phosphenes and disruption of motion perception can be elicited by TMS [93]. The coil was held tangentially to the scalp with the handle pointing upward and laterally at 45° angle to the sagittal plane. To target V1/V2, the coil was centered 2 cm dorsal to the inion, corresponding to the scalp position where phosphenes in the center of the visual field are typically elicited. The handle was held tangentially to the scalp and pointed downward at an angle of 120° clockwise. On each pair, the first TMS pulse was delivered to the left V5/MT+ followed by another pulse, delivered over V1/V2 with an ISI of 20 ms. This ISI was selected in accordance with the average timing of V5/MT+-V1/V2 interactions reported by Pascual-Leone and Walsh (2001) and Silvanto and colleagues (2005) and corresponds to the optimal timing at which V5/MT+ exerts a physiological effect on V1/V2 [28,94]. This ISI was critical to repeatedly activate presynaptic and postsynaptic neurons in reentrant V5/MT+-V1/V2 connections in a way that is consistent with STDP, i.e., a form of synaptic plasticity meeting the Hebbian principle and predicting that synapses are potentiated if the presynaptic neuron fires repeatedly before the postsynaptic neuron. Thus, ccPAS in the Exp_*V5-V1*_ was aimed at strengthening reentrant connections from V5/MT+ to V1/V2 in order to affect accuracy.

#### Experimental ccPAS condition IPS/LIP-to-V1/V2 (Exp_IPS-V1_)

The first pulse was delivered to the left IPS/LIP area followed by another pulse, delivered over V1/V2 (S7 Fig). Considering that we want to stimulate the human homolog of the LIP area, anatomical and functional studies suggest that IPS is the critical area for this purpose [95–97]. For this reason, we selected as a proxy for the stimulation site the P3 EEG coordinate (International System 10 to 20), because it was coincident with the spatial positioning of IPS/LIP according to previous studies [98–100]. V1/V2 was targeted as in the other experimental group. Temporal sequence of stimulation was set to 30 ms, in accordance with the average timing of interaction at which IPS/LIP exerts a physiological effect on V1/V2 [50,51]. This ISI adopted in Exp_*IPS-V1*_ was thus critical to enhance reentrant IPS/LIP-to-V1/V2 connections according to STDP [37].

#### Control condition IPS/LIP—V1/V2 (Ctrl_IPS-V1_)

In this condition, the target areas were the same as in the *Exp*_*IPS-V1*_; however, the pulses were released on both areas simultaneously (ISI = 0). In accordance with Hebbian principles [101], reinforcing synapses requires temporally asynchronous activations, so in the circumstance in which 2 neurons are activated at the same time, the precondition for enhancing the efficiency of their connection is not satisfied. Consequently, no STDP is expected following such control ccPAS manipulation [45,46]. Yet, this protocol was necessary to assess whether any change obtained at the behavioral level following Exp_IPS-V1_ was generated by mere stimulation of parieto-occipital areas, independently of the particular protocol applied.

### Data analysis

#### Motion sensitivity threshold

Discrimination performances collected through the task were plotted on a cartesian plane with the X axis representing the motion coherence and the Y axis the percentage of accuracy (S1–S3 Figs for individual plots). Data distribution described a psychophysical curve having a sigmoidal shape roughly ranging between 50% (at 0% of motion coherence; guessing rate) and 100% (at 80% of motion coherence) of accuracy. Therefore, data were well fitted by a nonlinear function modeled on the logistic curve:

$$y=\frac{1}{2}\left(1+\frac{a}{1+\exp(-\frac{x-b}{c})}\right)$$

where “a” determines the value of the upper horizontal asymptote; “b” represents the value of the point of critical change in the function behavior at half the way between the lower and the upper asymptotes, named the inflection point (IP) of the curve; and “c” defines the slope. For each participant, all parameters of the curve for each block were calculated using MATLAB (version 2019b, the MathWorks, Natick, MA), applying the Levenberg–Marquardt algorithm. By solving the equation, we defined the exact value representing the motion sensitivity threshold as the coherence level at which the direction was correctly perceived 75% of the times. This was intended as the percentage of coherent motion that mathematically described the change in the global motion perception. Shifts in motion sensitivity threshold were baseline corrected such that the values obtained in the performance at each time after the stimulation were subtracted from the value obtained in the performance at baseline. In this way, any negative value reflected enhancement in performance, while positive values reflected reduction in performance, compared to baseline values. The value of the R^2^ was calculated to verify the goodness of fit of individual’s data points to the logistic curve (Table A in S1 File).

#### Metacognition

As a measure of the relative perceptual awareness about the response on the first-order performance (i.e., direction of the stimulus), we estimated the metacognitive efficiency, which represents the optimality with which confidence ratings discriminate between “correct” and “incorrect” trials, while controlling for differences in perceptual sensitivity [2,61]. Since the efficiency measure considers the extent to which metacognitively optimal observers are aware of their performance [102], we used this as a proxy for awareness in metacognition, consistently with prior studies [103,104]. We adopted a single-subject Bayesian estimation approach, which is more robust to low trial numbers and does not use correction for missing cases relatively to previous implementation of meta-d’ [102]. We considered the metacognitive efficiency scores (i.e., meta-d’- d’) a reliable measure of second-order performance since they are not biased by differences in perceptual sensitivity between conditions as meta-d’ per se [105]. In reporting confidence, an observer is “metacognitively ideal” (meta-d’ = d’) when employing all of the sensory knowledge accessible for the type 1 choice; negative values point to suboptimality (meta-d’<d’), whereas positive values mean hyper-metacognitive evaluation (meta-d’>d’). After obtaining these estimates, performance change was obtained by subtracting the baseline values from the poststimulation sessions, resulting in positive values for metacognitive gain and negative ones for metacognition reduction. Finally, we analyzed metacognitive performance at threshold level by considering, for each subject in each session, the mean values of metacognitive efficiency of the 2 coherence levels containing the actual motion sensitivity threshold (e.g., for a motion threshold of approximately 9%, the resulting metacognition was averaged from the 8% and 12% coherence levels). This approach was consistent with prior works [12,80,103], which examined metacognition at the closest contrast to the participants’ detection threshold.

#### Statistical tests

To assess the effect of the ccPAS condition on discrimination performance at the coherent motion task, a repeated measure ANOVA with Targeted Network (Exp_*V5-V1*_; Exp_*IPS-V1;*_ *Ctrl*_*IPS-V1*_) as between-subject factor, and Time (T0, T30) as within-subject factor was performed on baseline-corrected motion threshold. To evaluate the effect of the ccPAS on metacognition, a repeated-measure ANOVA including the Targeted Network (Exp_*V5-V1*_; Exp_*IPS-V1*_*; Ctrl*_*IPS-V1*_) as a between-subject factor, and Time (T0, T30) as within-subject factors was performed on baseline-corrected metacognitive efficiency (i.e., dependent variable). Post hoc analysis was performed using the Duncan test to correct for multiple comparisons. Bonferroni–Holm corrected *t* test was performed for one-sample comparison in conditions of main interest.

In all analyses, effect sizes were estimated by Cohen’s d or ηp2. All data distributions were subjected to visual inspection to assess normality. All frequentist analyses were implemented using STATISTICA v.12 and MATLAB.

Bayesian repeated measure ANOVAs were implemented in the main analyses for sensitivity and metacognition to evaluate the strengths of evidence for the null and alternative hypothesis by computing the model-averaged results. The inclusion Bayes factor (i.e., BF_inclusion)_ for matched models was estimated. This quantifies the change from prior inclusion odds to posterior inclusion odds and can be interpreted as the evidence in the data for including a predictor in a model [106]. Bayesian one-sample *t* test was additionally performed on averaged baseline-corrected values for sensitivity and confidence, comparing the null model H0, which posits that the effect size δ is zero, to the alternative hypothesis H1 [107]. The Bayes factor was obtained by setting default t-prior [108]. All Bayesian analyses were implemented by the JASP software [109].

## Supporting information

S1 Fig ExpV5-V1 individual psychometric curves. Performance before (baseline: BSL black dotted line), immediately after (T0: red dashed line) and 30 minutes after Exp_V5-V1_ ccPAS (T30: red line). Perceptual thresholds (gray dots) shifts on the abscissa represent lower (right-shift) or higher (left-shift) motion sensitivity. Data underlying this figure can be found in OSF: https://osf.io/x7d2e/?view_only=ac2ff19b1ab6415cb471895854fb5a35. BSL, baseline; ccPAS, corticocortical paired associative stimulation; IP, inflection point.
(TIF)

S2 Fig ExpIPS-V1 individual psychometric curves. Performance before (baseline: BSL black dotted line), immediately after (T0: red dashed line) and 30 minutes after Exp_IPS-V1_ ccPAS (T30: red line). Perceptual thresholds (gray dots) shifts on the abscissa represent lower (right-shift) or higher (left-shift) motion sensitivity. Data underlying this figure can be found in OSF: https://osf.io/x7d2e/?view_only=ac2ff19b1ab6415cb471895854fb5a35. BSL, baseline; ccPAS, corticocortical paired associative stimulation; IP, inflection point.
(TIF)

S3 Fig CtrlIPS-V1 individual psychometric curves. Performance before (baseline: BSL black dotted line), immediately after (T0: red dashed line) and 30 minutes after Ctrl_IPS-V1_ ccPAS (T30: red line). Perceptual thresholds (gray dots) shifts on the abscissa represent lower (right-shift) or higher (left-shift) motion sensitivity. Data underlying this figure can be found in OSF: https://osf.io/x7d2e/?view_only=ac2ff19b1ab6415cb471895854fb5a35. BSL, baseline; ccPAS, corticocortical paired associative stimulation; IP, inflection point.
(TIF)

S4 Fig Raw psychometric threshold across sessions. Perceptual threshold values for each condition. Filled bars and dots represent mean and individual subject performances, respectively. Lower values indicate higher motion sensitivity. Data underlying this figure can be found in OSF: https://osf.io/x7d2e/?view_only=ac2ff19b1ab6415cb471895854fb5a35.
(TIF)

S5 Fig Raw metacognitive efficiency across sessions. Negative values of efficiency represent suboptimal metacognition (meta-d’<d’), positive values indicate “hyper” metacognition (meta-d’>d’), whereas null values (meta-d’ = d’) means “ideal” metacognition. Filled bars and dots represent mean and individual subject performances, respectively. Data underlying this figure can be found in OSF: https://osf.io/x7d2e/?view_only=ac2ff19b1ab6415cb471895854fb5a35.
(TIF)

S6 Fig Perceptual confidence as a function of performance. Average post-ccPAS confidence level as a function of correct (left side) and incorrect (right side) first-order performance. Filled bars represent mean (T0+T30) poststimulation values, and dots show individual subject performances. Asterisks point to significant *p* < .05 corrected one-sample *t* test. Data underlying this figure can be found in OSF: https://osf.io/x7d2e/?view_only=ac2ff19b1ab6415cb471895854fb5a35.
(TIF)

S7 Fig Cortical projections of TMS sites. Single subjects’ coordinates of stimulation site for (**a)** V5/MT+ (red) and V1/V2 (black) and (**b)** IPS/LIP (blue) and V1/V2 (black), projected on a rendered brain surface from geometrical and EEG scalp positions reconstructed using MRICro (https://www.nitrc.org/projects/mricro).
(TIF)

S1 File Supporting results and methods. Text A. Additional analysis on behavioral measure relative to first-order performance. Text B. Additional analysis on second-order performance. Text C. Supplementary discussion. Text D. Supplementary methods. Table A. Mean values ± SEM for hit rate (%), false alarm rate (%), criterion, and reaction times (ms) at each session, separately for condition. Table B. Mean R2 and slope ± SEM for each session, separately for condition.
(DOCX)

## Abbreviations:

BSL: baseline
ccPAS: corticocortical paired associative stimulation
IP: inflection point
IPS: intraparietal sulcus
LIP: lateral intraparietal cortex
STDP: spike timing–dependent plasticity
TMS: transcranial magnetic stimulation

## References

[1] Yeung N, Summerfield C. Metacognition in human decision-making: Confidence and error monitoring. Philos Trans R Soc Lond B Biol Sci. 2012;367:1310–1321. doi: 10.1098/rstb.2011.0416 22492749 PMC3318764

[2] Fleming SM, Daw ND. Self-evaluation of decision-making: A general bayesian framework for metacognitive computation. Psychol Rev. 2017;124:91–114. doi: 10.1037/rev0000045 28004960 PMC5178868

[3] Maniscalco B, Peters MAK, Lau H. Heuristic use of perceptual evidence leads to dissociation between performance and metacognitive sensitivity. Atten Percept Psychophys. 2016;78:923–937. doi: 10.3758/s13414-016-1059-x 26791233 PMC4811689

[4] Rahnev DA, Maniscalco B, Luber B, Lau H, Lisanby SH. Direct injection of noise to the visual cortex decreases accuracy but increases decision confidence. J Neurophysiol. 2012;107:1556–1563. doi: 10.1152/jn.00985.2011 22170965

[5] Di Gregorio F, Trajkovic J, Roperti C, Marcantoni E, Di Luzio P, Avenanti A, et al. Tuning alpha rhythms to shape conscious visual perception. Curr Biol. 2022;32:988–998.e6. doi: 10.1016/j.cub.2022.01.003 35090592

[6] Ferrigno S, Kornell N, Cantlon JF. A metacognitive illusion in monkeys. Proc Biol Sci. 2017;284. doi: 10.1098/rspb.2017.1541 28878068 PMC5597844

[7] Boldt A, de Gardelle V, Yeung N. The impact of evidence reliability on sensitivity and bias in decision confidence. J Exp Psychol Hum Percept Perform. 2017;43:1520–1531. doi: 10.1037/xhp0000404 28383959 PMC5524444

[8] Zylberberg A, Barttfeld P, Sigman M. The construction of confidence in a perceptual decision. Front Integr Neurosci. 2012;6:79. doi: 10.3389/fnint.2012.00079 23049504 PMC3448113

[9] Samaha J, Barrett JJ, Sheldon AD, LaRocque JJ, Postle BR. Dissociating Perceptual Confidence from Discrimination Accuracy Reveals No Influence of Metacognitive Awareness on Working Memory. Front Psychol. 2016;7:851. doi: 10.3389/fpsyg.2016.00851 27375529 PMC4893488

[10] Vlassova A, Donkin C, Pearson J. Unconscious information changes decision accuracy but not confidence. Proc Natl Acad Sci U S A. 2014;111:16214–16218. doi: 10.1073/pnas.1403619111 25349435 PMC4234611

[11] Fleming SM, Maniscalco B, Ko Y, Amendi N, Ro T, Lau H. Action-Specific Disruption of Perceptual Confidence. Psychol Sci. 2015;26:89–98. doi: 10.1177/0956797614557697 25425059 PMC4361353

[12] Rounis E, Maniscalco B, Rothwell JC, Passingham RE, Lau H. Theta-burst transcranial magnetic stimulation to the prefrontal cortex impairs metacognitive visual awareness. Cogn Neurosci. 2010;1:165–175. doi: 10.1080/17588921003632529 24168333

[13] Rahnev D, Koizumi A, McCurdy LY, D’Esposito M, Lau H. Confidence Leak in Perceptual Decision Making. Psychol Sci. 2015;26:1664–1680. doi: 10.1177/0956797615595037 26408037 PMC4636919

[14] Allen M, Frank D, Samuel Schwarzkopf D, Fardo F, Winston JS, Hauser TU, et al. Unexpected arousal modulates the influence of sensory noise on confidence. Elife. 2016;5. doi: 10.7554/eLife.18103 27776633 PMC5079750

[15] Maniscalco B, McCurdy LY, Odegaard B, Lau H. Limited Cognitive Resources Explain a Trade-Off between Perceptual and Metacognitive Vigilance. J Neurosci. 2017;37:1213–1224. doi: 10.1523/JNEUROSCI.2271-13.2016 28028197 PMC6596853

[16] Peters MAK, Thesen T, Ko YD, Maniscalco B, Carlson C, Davidson M, et al. Perceptual confidence neglects decision-incongruent evidence in the brain. Nat Hum Behav. 2017;1:1–8. doi: 10.1038/s41562-017-0139 29130070 PMC5675133

[17] Samaha J, Iemi L, Postle BR. Prestimulus alpha-band power biases visual discrimination confidence, but not accuracy. Conscious Cogn. 2017;54:47–55. doi: 10.1016/j.concog.2017.02.005 28222937 PMC5561529

[18] Hanks TD, Summerfield C. Perceptual Decision Making in Rodents, Monkeys, and Humans. Neuron. 2017;93:15–31. doi: 10.1016/j.neuron.2016.12.003 28056343

[19] Britten KH, Shadlen MN, Newsome WT, Movshon JA. The analysis of visual motion: a comparison of neuronal and psychophysical performance. J Neurosci. 1992;12:4745–4765. doi: 10.1523/JNEUROSCI.12-12-04745.1992 1464765 PMC6575768

[20] Ditterich J, Mazurek ME, Shadlen MN. Microstimulation of visual cortex affects the speed of perceptual decisions. Nat Neurosci. 2003;6:891–898. doi: 10.1038/nn1094 12858179

[21] Fetsch CR, Kiani R, Newsome WT, Shadlen MN. Effects of Cortical Microstimulation on Confidence in a Perceptual Decision. Neuron. 2014;83:797–804. doi: 10.1016/j.neuron.2014.07.011 25123306 PMC4141901

[22] Hanks TD, Ditterich J, Shadlen MN. Microstimulation of macaque area LIP affects decision-making in a motion discrimination task. Nat Neurosci. 2006;9:682–689. doi: 10.1038/nn1683 16604069 PMC2770004

[23] Kiani R, Shadlen MN. Representation of confidence associated with a decision by neurons in the parietal cortex. Science. 2009;324:759–764. doi: 10.1126/science.1169405 19423820 PMC2738936

[24] Heekeren HR, Marrett S, Ungerleider LG. The neural systems that mediate human perceptual decision making. Nat Rev Neurosci. 2008;9:467–479. doi: 10.1038/nrn2374 18464792

[25] Gould IC, Nobre AC, Wyart V, Rushworth MFS. Effects of decision variables and intraparietal stimulation on sensorimotor oscillatory activity in the human brain. J Neurosci. 2012;32:13805–13818. doi: 10.1523/JNEUROSCI.2200-12.2012 23035092 PMC3491879

[26] Gold JI, Shadlen MN. The Neural Basis of Decision Making. Annu Rev Neurosci. 2007;30:535–574. doi: 10.1146/annurev.neuro.29.051605.113038 17600525

[27] Zhou Y, Freedman DJ. Posterior parietal cortex plays a causal role in perceptual and categorical decisions. Science. 2019;365:180–185. doi: 10.1126/science.aaw8347 31296771 PMC7346736

[28] Silvanto J, Lavie N, Walsh V. Double Dissociation of V1 and V5/MT activity in Visual Awareness. Cereb Cortex. 2005;15:1736–1741. doi: 10.1093/cercor/bhi050 15703247

[29] Vetter P, Grosbras M-H, Muckli L. TMS Over V5 Disrupts Motion Prediction. Cereb Cortex. 2015;25:1052–1059. doi: 10.1093/cercor/bht297 24152544 PMC4380002

[30] Suppa A, Quartarone A, Siebner H, Chen R, Di Lazzaro V, Del Giudice P, et al. The associative brain at work: Evidence from paired associative stimulation studies in humans. Clin Neurophysiol. 2017;128:2140–2164. doi: 10.1016/j.clinph.2017.08.003 28938144

[31] Rizzo V, Siebner HS, Morgante F, Mastroeni C, Girlanda P, Quartarone A. Paired Associative Stimulation of Left and Right Human Motor Cortex Shapes Interhemispheric Motor Inhibition based on a Hebbian Mechanism. Cereb Cortex. 2009;19:907–915. doi: 10.1093/cercor/bhn144 18791179

[32] Romei V, Thut G, Silvanto J. Information-Based Approaches of Noninvasive Transcranial Brain Stimulation. Trends Neurosci. 2016;39:782–795. doi: 10.1016/j.tins.2016.09.001 27697295

[33] Guidali G, Roncoroni C, Bolognini N. Paired associative stimulations: Novel tools for interacting with sensory and motor cortical plasticity. Behav Brain Res. 2021;414:113484. doi: 10.1016/j.bbr.2021.113484 34302877

[34] Pitcher D, Parkin B, Walsh V. Transcranial Magnetic Stimulation and the Understanding of Behavior. Annu Rev Psychol. 2021;72:97–121. doi: 10.1146/annurev-psych-081120-013144 33095690

[35] Jackson A, Mavoori J, Fetz EE. Long-term motor cortex plasticity induced by an electronic neural implant. Nature. 2006;444:56–60. doi: 10.1038/nature05226 17057705

[36] Caporale N, Dan Y. Spike Timing–Dependent Plasticity: A Hebbian Learning Rule. Annu Rev Neurosci. 2008;31: 25–46. doi: 10.1146/annurev.neuro.31.060407.125639 18275283

[37] Markram H, Gerstner W, Sjöström PJ. Spike-timing-dependent plasticity: A comprehensive overview. Front Synaptic Neurosci. 2012;4:2. doi: 10.3389/fnsyn.2012.00002 22807913 PMC3395004

[38] Arai N, Lu M-K, Ugawa Y, Ziemann U. Effective connectivity between human supplementary motor area and primary motor cortex: a paired-coil TMS study. Exp Brain Res. 2012;220:79–87. doi: 10.1007/s00221-012-3117-5 22623093

[39] Buch ER, Johnen VM, Nelissen N, Shea JO, Rushworth MFS. Noninvasive Associative Plasticity Induction in a Corticocortical Pathway of the Human Brain. J Neurosci. 2011;31:17669–17679. doi: 10.1523/JNEUROSCI.1513-11.2011 22131427 PMC6623800

[40] Chiappini E, Borgomaneri S, Marangon M, Turrini S, Romei V, Avenanti A. Driving associative plasticity in premotor-motor connections through a novel paired associative stimulation based on long-latency cortico-cortical interactions. Brain Stimul. 2020;13:1461–1463. doi: 10.1016/j.brs.2020.08.003 32791314

[41] Veniero D, Ponzo V, Koch G. Paired Associative Stimulation Enforces the Communication between Interconnected Areas. J Neurosci. 2013;33:13773–13783. doi: 10.1523/JNEUROSCI.1777-13.2013 23966698 PMC6618662

[42] Rizzo V, Bove M, Naro A, Tacchino A, Mastroeni C, Avanzino L, et al. Associative cortico-cortical plasticity may affect ipsilateral finger opposition movements. Behav Brain Res. 2011;216:433–439. doi: 10.1016/j.bbr.2010.08.037 20816702

[43] Fiori F, Chiappini E, Avenanti A. Enhanced action performance following TMS manipulation of associative plasticity in ventral premotor-motor pathway. Neuroimage. 2018;183:847–858. doi: 10.1016/j.neuroimage.2018.09.002 30193973

[44] Chiappini E, Silvanto J, Hibbard PB, Avenanti A, Romei V. Strengthening functionally specific neural pathways with transcranial brain stimulation. Curr Biol. 2018;28:R735–R736. doi: 10.1016/j.cub.2018.05.083 29990453

[45] Romei V, Chiappini E, Hibbard PB, Avenanti A. Empowering Reentrant Projections from V5 to V1 Boosts Sensitivity to Motion. Curr Biol. 2016;26:2155–2160. doi: 10.1016/j.cub.2016.06.009 27524488

[46] Gherman S, Philiastides MG. Neural representations of confidence emerge from the process of decision formation during perceptual choices. Neuroimage. 2015;106:134–143. doi: 10.1016/j.neuroimage.2014.11.036 25463461

[47] Greenberg AS, Verstynen T, Chiu Y-C, Yantis S, Schneider W, Behrmann M. Visuotopic Cortical Connectivity Underlying Attention Revealed with White-Matter Tractography. J Neurosci. 2012;32:2773–2782. doi: 10.1523/JNEUROSCI.5419-11.2012 22357860 PMC3321828

[48] Lewis JW, Van Essen DC. Corticocortical Connections of Visual, Sensorimotor, and Multimodal Processing Areas in the Parietal Lobe of the Macaque Monkey. J Comp Neurol. 2000;428:112–137. doi: 10.1002/1096-9861(20001204)428:1&lt;112::aid-cne8&gt;3.0.co;2-9 11058227

[49] Baizer JS, Ungerleider LG, Desimone R. Organization of visual inputs to the inferior temporal and posterior parietal cortex in macaques. J Neurosci. 1991;11:168–190. doi: 10.1523/JNEUROSCI.11-01-00168.1991 1702462 PMC6575184

[50] Parks NA, Mazzi C, Tapia E, Savazzi S, Fabiani M, Gratton G, et al. The influence of posterior parietal cortex on extrastriate visual activity: A concurrent TMS and fast optical imaging study. Neuropsychologia. 2015;78:153–158. doi: 10.1016/j.neuropsychologia.2015.10.002 26449990 PMC4734125

[51] Silvanto J, Muggleton N, Lavie N, Walsh V. The perceptual and functional consequences of parietal top-down modulation on the visual cortex. Cereb Cortex. 2009;19:327–330. doi: 10.1093/cercor/bhn091 18515296 PMC2638787

[52] Limbach K, Corballis PM. Prestimulus alpha power influences response criterion in a detection task. Psychophysiology. 2016;53:1154–1164. doi: 10.1111/psyp.12666 27144476

[53] Ratcliff R, Starns JJ. Modeling Confidence Judgments, Response Times, and Multiple Choices in Decision Making: Recognition Memory and Motion Discrimination. Psychol Rev. 2013;120:697. doi: 10.1037/a0033152 23915088 PMC4106127

[54] De Martino B, Fleming SM, Garrett N, Dolan RJ. Confidence in value-based choice. Nat Neurosci. 2013;16:105–110. doi: 10.1038/nn.3279 23222911 PMC3786394

[55] Rahnev D, Nee DE, Riddle J, Larson AS, D’Esposito M. Causal evidence for frontal cortex organization for perceptual decision making. Proc Natl Acad Sci. 2016;113:6059–6064. doi: 10.1073/pnas.1522551113 27162349 PMC4889369

[56] Jang Y, Wallsten TS, Huber DE. A stochastic detection and retrieval model for the study of metacognition. Psychol Rev. 2012;119:186–200. doi: 10.1037/a0025960 22059901

[57] Yeon J, Shekhar M, Rahnev D. Overlapping and unique neural circuits are activated during perceptual decision making and confidence. Sci Rep. 2020;10:1–13. doi: 10.1038/s41598-020-77820-6 33247212 PMC7699640

[58] Spence ML, Mattingley JB, Dux PE. Uncertainty information that is irrelevant for report impacts confidence judgments. J Exp Psychol Hum Percept Perform. 2018;44:1981–1994. doi: 10.1037/xhp0000584 30475052

[59] Bang JW, Shekhar M, Rahnev D. Sensory noise increases metacognitive efficiency. J Exp Psychol Gen. 2019;148:437–452. doi: 10.1037/xge0000511 30382720

[60] Wokke ME, Cleeremans A, Ridderinkhof KR. Sure I’m Sure: Prefrontal Oscillations Support Metacognitive Monitoring of Decision Making. J Neurosci. 2017;37:781–789. doi: 10.1523/JNEUROSCI.1612-16.2016 28123015 PMC6597021

[61] Maniscalco B, Lau H. A signal detection theoretic approach for estimating metacognitive sensitivity from confidence ratings. Conscious Cogn. 2012;21:422–430. doi: 10.1016/j.concog.2011.09.021 22071269

[62] Sillito AM, Cudeiro J, Jones HE. Always returning: feedback and sensory processing in visual cortex and thalamus. Trends Neurosci. 2006;29:307–316. doi: 10.1016/j.tins.2006.05.001 16713635

[63] Shekhar M, Rahnev D. The nature of metacognitive inefficiency in perceptual decision making. Psychol Rev. 2021;128:45–70. doi: 10.1037/rev0000249 32673034 PMC7883626

[64] Shekhar M, Rahnev D. Distinguishing the Roles of Dorsolateral and Anterior PFC in Visual Metacognition. J Neurosci. 2018;38:5078–5087. doi: 10.1523/JNEUROSCI.3484-17.2018 29720553 PMC6705938

[65] Vogels TP, Abbott LF. Gating multiple signals through detailed balance of excitation and inhibition in spiking networks. Nat Neurosci. 2009;12:483–491. doi: 10.1038/nn.2276 19305402 PMC2693069

[66] Michalareas G, Vezoli J, van Pelt S, Schoffelen JM, Kennedy H, Fries P. Alpha-Beta and Gamma Rhythms Subserve Feedback and Feedforward Influences among Human Visual Cortical Areas. Neuron. 2016;89:384–397. doi: 10.1016/j.neuron.2015.12.018 26777277 PMC4871751

[67] Van Kerkoerle T, Self MW, Dagnino B, Gariel-Mathis MA, Poort J, Van Der Togt C, et al. Alpha and gamma oscillations characterize feedback and feedforward processing in monkey visual cortex. Proc Natl Acad Sci U S A. 2014;111:14332–14341. doi: 10.1073/pnas.1402773111 25205811 PMC4210002

[68] Nassi JJ, Lomber SG, Born RT. Corticocortical feedback contributes to surround suppression in V1 of the alert primate. J Neurosci. 2013;33:8504–8517. doi: 10.1523/JNEUROSCI.5124-12.2013 23658187 PMC3690087

[69] Shao Z, Burkhalter A. Different balance of excitation and inhibition in forward and feedback circuits of rat visual cortex. J Neurosci. 1996;16:7353–7365. doi: 10.1523/JNEUROSCI.16-22-07353.1996 8929442 PMC6578929

[70] Roelfsema PR, Lamme VAF, Spekreijse H, Bosch H. Figure-ground segregation in a recurrent network architecture. J Cogn Neurosci. 2002;14:525–537. doi: 10.1162/08989290260045756 12126495

[71] Hupé JM, James AC, Payne BR, Lomber SG, Girard P, Bullier J. Cortical feedback improves discrimination between figure and background by V1, V2 and V3 neurons. Nature. 1998;394:784–787. doi: 10.1038/29537 9723617

[72] Bevilacqua M, Huxlin KR, Hummel FC, Raffin E. Pathway and directional specificity of Hebbian plasticity induction in the cortical visual motion processing network. bioRxiv [Preprint]. 2022. Available from: https://www.biorxiv.org/content/10.1101/2022.05.15.491882v1.full 10.1016/j.isci.2023.107064 PMC10319215 37408682

[73] Gilbert CD, Li W. Top-down influences on visual processing. Nat Rev Neurosci. 2013;14:350–363. doi: 10.1038/nrn3476 23595013 PMC3864796

[74] Bach DR, Dolan RJ. Knowing how much you don’t know: A neural organization of uncertainty estimates. Nat Rev Neurosci. 2012;13:572–586. doi: 10.1038/nrn3289 22781958

[75] Summerfield C, de Lange FP. Expectation in perceptual decision making: neural and computational mechanisms. Nat Rev Neurosci. 2014;15:745–756. doi: 10.1038/nrn3838 25315388

[76] Haefner RM, Berkes P, Fiser J. Perceptual Decision-Making as Probabilistic Inference by Neural Sampling. Neuron. 2016;90:649–660. doi: 10.1016/j.neuron.2016.03.020 27146267

[77] Wilming N, Murphy PR, Meyniel F, Donner TH. Large-scale dynamics of perceptual decision information across human cortex. Nat Commun. 2020;11:5109. doi: 10.1038/s41467-020-18826-6 33037209 PMC7547662

[78] Murphy PR, Wilming N, Hernandez-Bocanegra DC, Prat-Ortega G, Donner TH. Adaptive circuit dynamics across human cortex during evidence accumulation in changing environments. Nat Neurosci. 2021;24:987–997. doi: 10.1038/s41593-021-00839-z 33903770

[79] Wimmer K, Compte A, Roxin A, Peixoto D, Renart A, De La Rocha J. Sensory integration dynamics in a hierarchical network explains choice probabilities in cortical area MT. Nat Commun. 2015;6:1–13. doi: 10.1038/ncomms7177 25649611 PMC4347303

[80] Bor D, Schwartzman DJ, Barrett AB, Seth AK. Theta-burst transcranial magnetic stimulation to the prefrontal or parietal cortex does not impair metacognitive visual awareness. PLoS ONE. 2017;12:1–20. doi: 10.1371/journal.pone.0171793 PMC5305100 28192502

[81] Rahnev D, Kok P, Munneke M, Bahdo L, de Lange FP, Lau H. Continuous theta burst transcranial magnetic stimulation reduces resting state connectivity between visual areas. J Neurophysiol. 2013;110:1811–1821. doi: 10.1152/jn.00209.2013 23883858

[82] Hurme M, Koivisto M, Revonsuo A, Railo H. Early processing in primary visual cortex is necessary for conscious and unconscious vision while late processing is necessary only for conscious vision in neurologically healthy humans. Neuroimage. 2017;150:230–238. doi: 10.1016/j.neuroimage.2017.02.060 28254455

[83] Qiu L, Su J, Ni Y, Bai Y, Zhang X, Li X, et al. The neural system of metacognition accompanying decision-making in the prefrontal cortex. PLoS Biol. 2018;16:e2004037. doi: 10.1371/journal.pbio.2004037 29684004 PMC5933819

[84] Cavada C, Goldman-Rakic PS. Posterior parietal cortex in rhesus monkey: II. Evidence for segregated corticocortical networks linking sensory and limbic areas with the frontal lobe. J Comp Neurol. 1989;287:422–445. doi: 10.1002/cne.902870403 2477406

[85] Andersen RA, Asanuma C, Essick G, Siegel RM. Corticocortical connections of anatomically and physiologically defined subdivisions within the inferior parietal lobule. J Comp Neurol. 1990;296:65–113. doi: 10.1002/cne.902960106 2358530

[86] Fleming SM, Huijgen J, Dolan RJ. Prefrontal contributions to metacognition in perceptual decision making. J Neurosci. 2012;32:6117–6125. doi: 10.1523/JNEUROSCI.6489-11.2012 22553018 PMC3359781

[87] Brainard DH. The Psychophysics Toolbox. Spat Vis. 1997;10:433–436. doi: 10.1163/156856897X00357 9176952

[88] Stefan K, Kunesch E, Cohen LG, Benecke R, Classen J. Induction of plasticity in the human motor cortex by paired associative stimulation. Brain. 2000;123(Pt 3):572–584. doi: 10.1093/brain/123.3.572 10686179

[89] Chao CC, Karabanov AN, Paine R, Carolina De Campos A, Kukke SN, Wu T, et al. Induction of motor associative plasticity in the posterior parietal cortex-primary motor network. Cereb Cortex. 2015;25:365–373. doi: 10.1093/cercor/bht230 23968834 PMC4303801

[90] Wolters A, Schmidt A, Schramm A, Zeller D, Naumann M, Kunesch E, et al. Timing-dependent plasticity in human primary somatosensory cortex. J Physiol. 2005;565:1039–1052. doi: 10.1113/jphysiol.2005.084954 15845584 PMC1464551

[91] Pitcher D, Walsh V, Yovel G, Duchaine B. TMS evidence for the involvement of the right occipital face area in early face processing. Curr Biol. 2007;17:1568–1573. doi: 10.1016/j.cub.2007.07.063 17764942

[92] Silvanto J, Muggleton NG, Cowey A, Walsh V. Neural activation state determines behavioral susceptibility to modified theta burst transcranial magnetic stimulation. Eur J Neurosci. 2007;26:523–528. doi: 10.1111/j.1460-9568.2007.05682.x 17650122

[93] Silvanto J, Muggleton NG. Testing the validity of the TMS state-dependency approach: Targeting functionally distinct motion-selective neural populations in visual areas V1/V2 and V5/MT+. Neuroimage. 2008;40:1841–1848. doi: 10.1016/j.neuroimage.2008.02.002 18353682

[94] Pascual-Leone A, Walsh V. Fast backprojections from the motion to the primary visual area necessary for visual awareness. Science. 2001;292:510–512. doi: 10.1126/science.1057099 11313497

[95] Connolly JD, Goodale MA, Menon RS, Munoz DP. Human fMRI evidence for the neural correlates of preparatory set. Nat Neurosci. 2002;5:1345–1352. doi: 10.1038/nn969 12411958

[96] Sereno MI, Pitzalis S, Martinez A. Mapping of contralateral space in retinotopic coordinates by a parietal cortical area in humans. Science. 2001;294:1350–1354. doi: 10.1126/science.1063695 11701930

[97] Schluppeck D, Glimcher P, Heeger DJ. Topographic organization for delayed saccades in human posterior parietal cortex. J Neurophysiol. 2005;94:1372–1384. doi: 10.1152/jn.01290.2004 15817644 PMC2367322

[98] Bagattini C, Mazzi C, Savazzi S. Waves of awareness for occipital and parietal phosphenes perception. Neuropsychologia. 2015;70:114–125. doi: 10.1016/j.neuropsychologia.2015.02.021 25698639

[99] Tapia E, Beck DM. Probing feedforward and feedback contributions to awareness with visual masking and transcranial magnetic stimulation. Front Psychol. 2014;5:1–14. doi: 10.3389/fpsyg.2014.01173 25374548 PMC4204434

[100] Mazzi C, Mancini F, Savazzi S. Can IPS reach visual awareness without V1? Evidence from TMS in healthy subjects and hemianopic patients. Neuropsychologia. 2014;64:134–144. doi: 10.1016/j.neuropsychologia.2014.09.026 25258247

[101] Hebb DO. The Organization of Behavior: A neuropsychological theory. Wiley; 1949.

[102] Fleming SM. HMeta-d: hierarchical Bayesian estimation of metacognitive efficiency from confidence ratings. Neurosci Conscious. 2017;2017:nix007. doi: 10.1093/nc/nix007 29877507 PMC5858026

[103] Lapate RC, Samaha J, Rokers B, Postle BR, Davidson RJ. Perceptual metacognition of human faces is causally supported by function of the lateral prefrontal cortex. Commun Biol. 2020;3. doi: 10.1038/s42003-020-1049-3 32647260 PMC7347936

[104] Palmer EC, David AS, Fleming SM. Effects of age on metacognitive efficiency. Conscious Cogn. 2014;28:151–160. doi: 10.1016/j.concog.2014.06.007 25064692 PMC4154452

[105] Beck B, Peña-Vivas V, Fleming S, Haggard P. Metacognition across sensory modalities: Vision, warmth, and nociceptive pain. Cognition. 2019;186:32–41. doi: 10.1016/j.cognition.2019.01.018 30739057 PMC6411924

[106] Van Den Bergh D, Van Doorn J, Marsman M, Draws T, Van Kesteren EJ, Derks K, et al. A tutorial on conducting and interpreting a bayesian ANOVA in JASP. Annee Psychol. 2020;120:73–96. doi: 10.3917/anpsy1.201.0073

[107] Wagenmakers EJ, Love J, Marsman M, Jamil T, Ly A, Verhagen J, et al. Bayesian inference for psychology. Part II: Example applications with JASP. Psychon Bull Rev. 2018;25:58–76. doi: 10.3758/s13423-017-1323-7 28685272 PMC5862926

[108] van Doorn J, van den Bergh D, Böhm U, Dablander F, Derks K, Draws T, et al. The JASP guidelines for conducting and reporting a Bayesian analysis. Psychon Bull Rev. 2021;28:813–826. doi: 10.3758/s13423-020-01798-5 33037582 PMC8219590

[109] JASP Team. JASP (Version 0.16.3) [Computer software]. 2022.

