# Supplementary figures and images for: Human perceptual and metacognitive decision-making rely on distinct brain networks
Source: PLoS Biol. 2022 Aug 9;20(8):e3001750. doi: 10.1371/journal.pbio.3001750 (PMC9362930; doi:10.1371/journal.pbio.3001750)

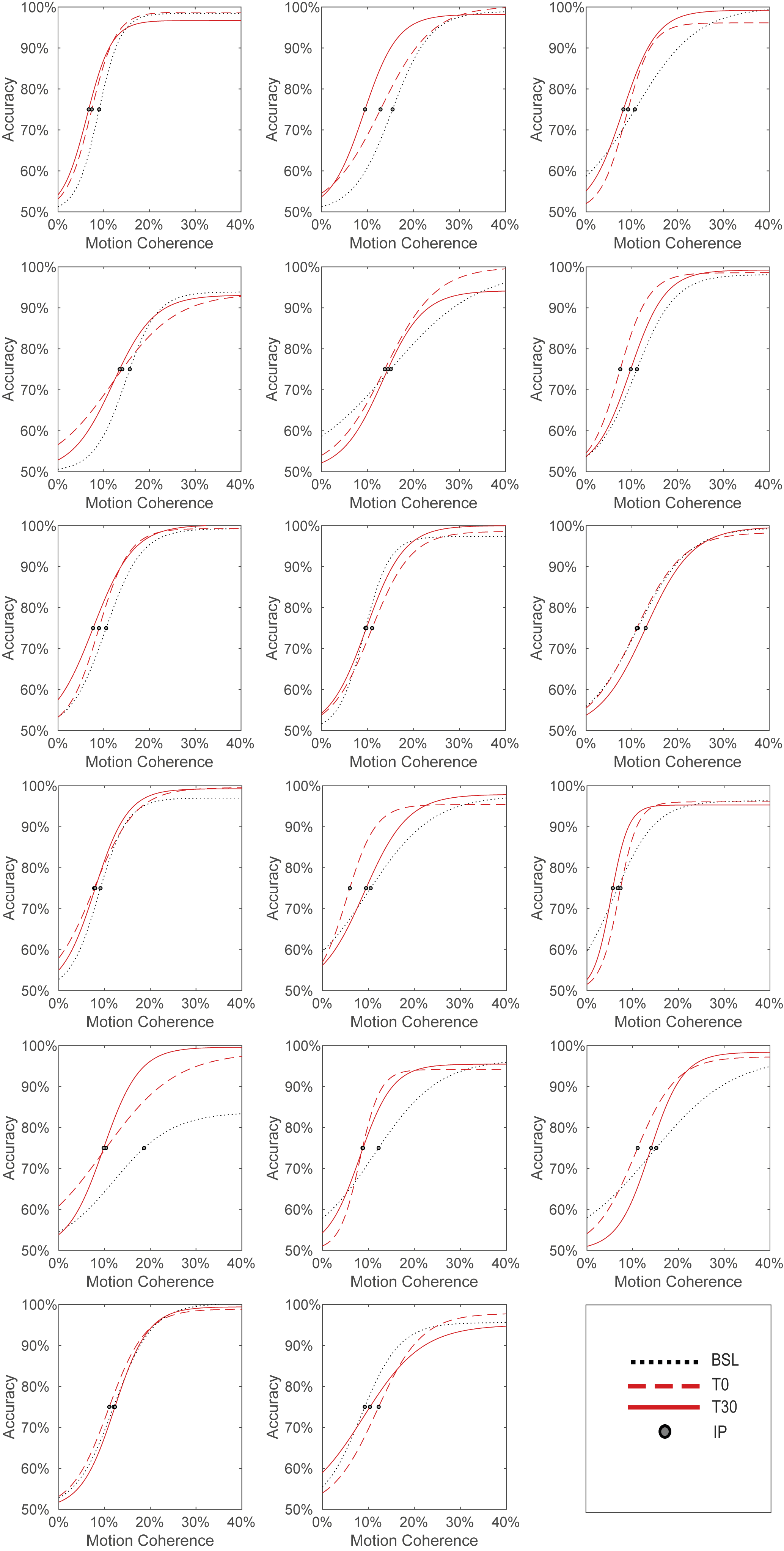

Supplement: S1 Fig — Performance before (baseline: BSL black dotted line), immediately after (T0: red dashed line) and 30 minutes after ExpV5-V1 ccPAS (T30: red line). Perceptual thresholds (gray dots) shifts on the abscissa represent lower (right-shift) or higher (left-shift) motion sensitivity. Data underlying this figure can be found in OSF: https://osf.io/x7d2e/?view_only=ac2ff19b1ab6415cb471895854fb5a35. BSL, baseline; ccPAS, corticocortical paired associative stimulation; IP, inflection point. (TIF) [file pbio.3001750.s001.tif]

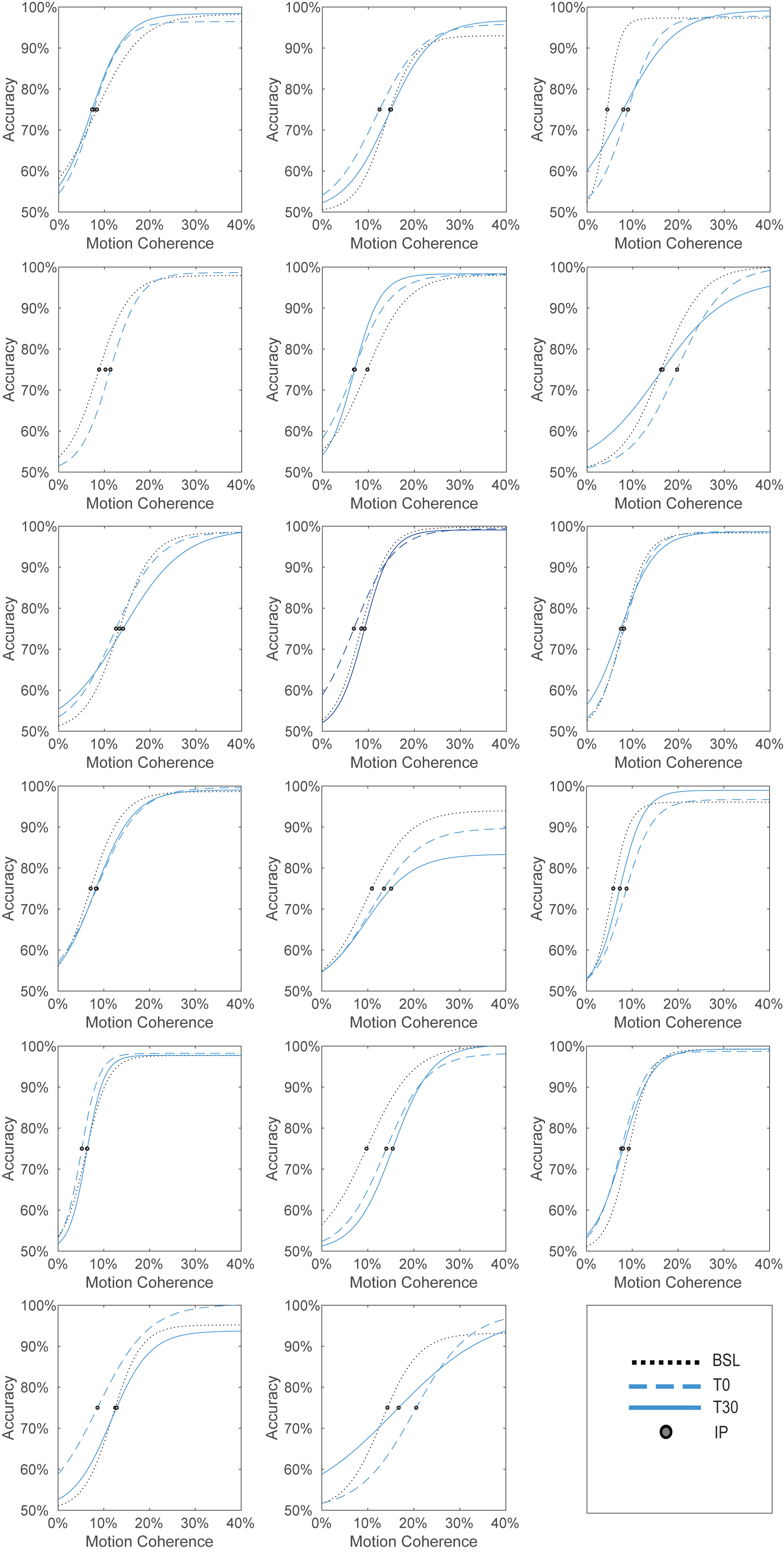

Supplement: S2 Fig — Performance before (baseline: BSL black dotted line), immediately after (T0: red dashed line) and 30 minutes after ExpIPS-V1 ccPAS (T30: red line). Perceptual thresholds (gray dots) shifts on the abscissa represent lower (right-shift) or higher (left-shift) motion sensitivity. Data underlying this figure can be found in OSF: https://osf.io/x7d2e/?view_only=ac2ff19b1ab6415cb471895854fb5a35. BSL, baseline; ccPAS, corticocortical paired associative stimulation; IP, inflection point. (TIF) [file pbio.3001750.s002.tif]

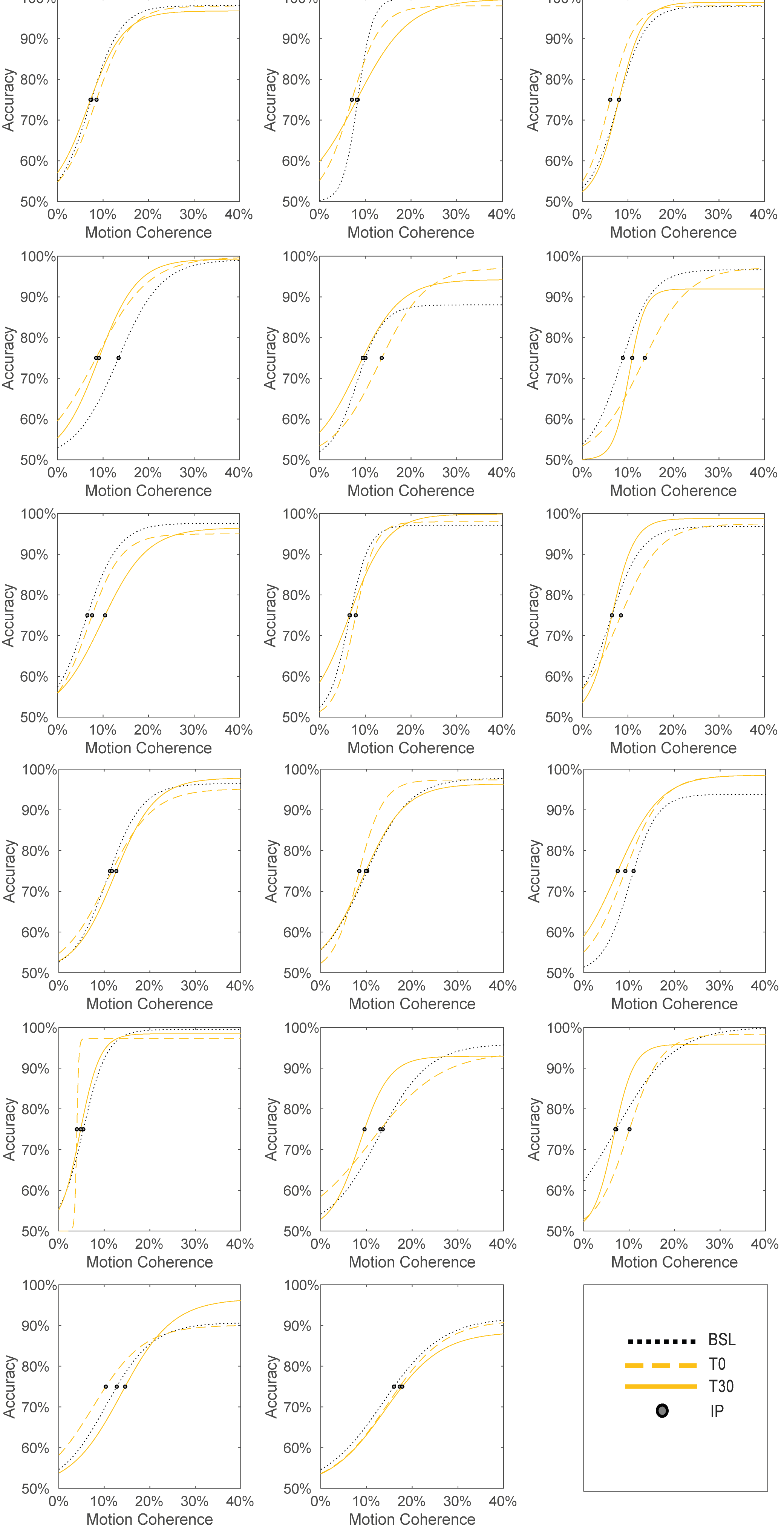

Supplement: S3 Fig — Performance before (baseline: BSL black dotted line), immediately after (T0: red dashed line) and 30 minutes after CtrlIPS-V1 ccPAS (T30: red line). Perceptual thresholds (gray dots) shifts on the abscissa represent lower (right-shift) or higher (left-shift) motion sensitivity. Data underlying this figure can be found in OSF: https://osf.io/x7d2e/?view_only=ac2ff19b1ab6415cb471895854fb5a35. BSL, baseline; ccPAS, corticocortical paired associative stimulation; IP, inflection point. (TIF) [file pbio.3001750.s003.tif]

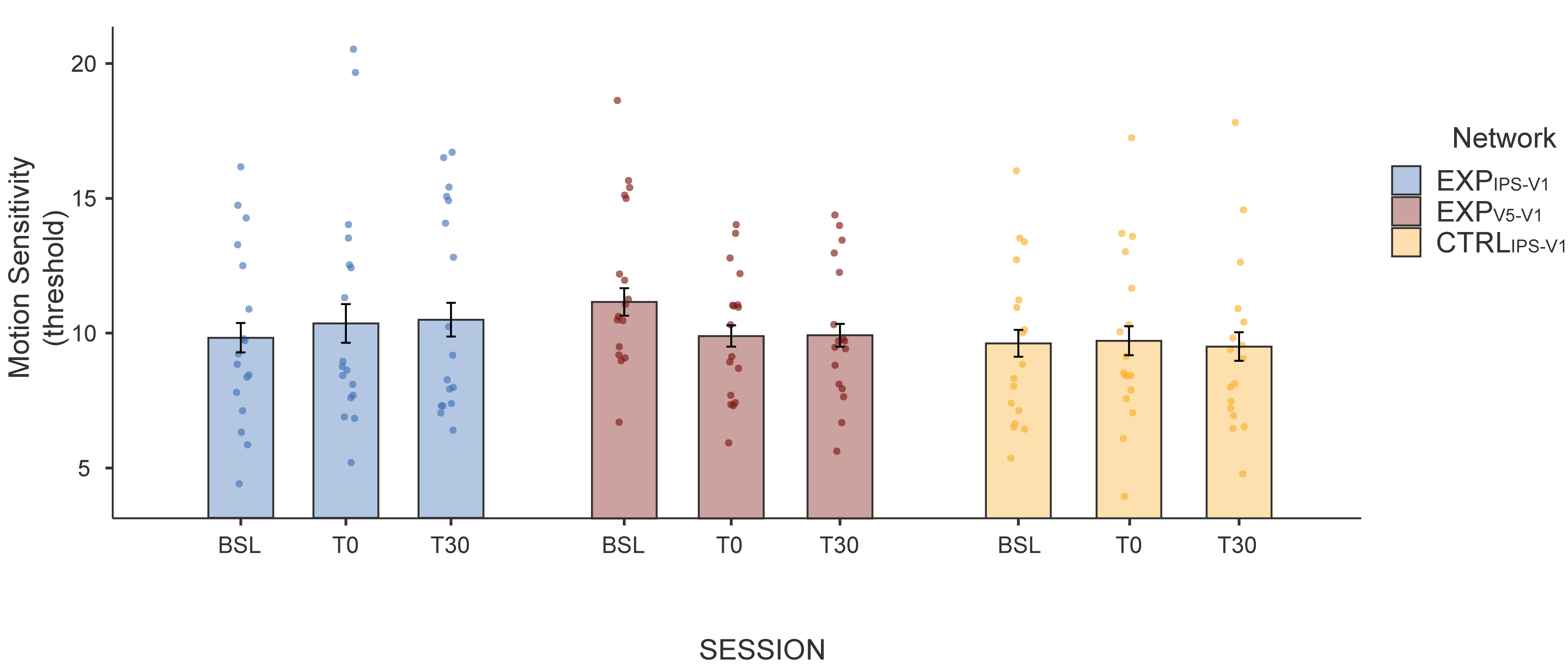

Supplement: S4 Fig — Perceptual threshold values for each condition. Filled bars and dots represent mean and individual subject performances, respectively. Lower values indicate higher motion sensitivity. Data underlying this figure can be found in OSF: https://osf.io/x7d2e/?view_only=ac2ff19b1ab6415cb471895854fb5a35. (TIF) [file pbio.3001750.s004.tif]

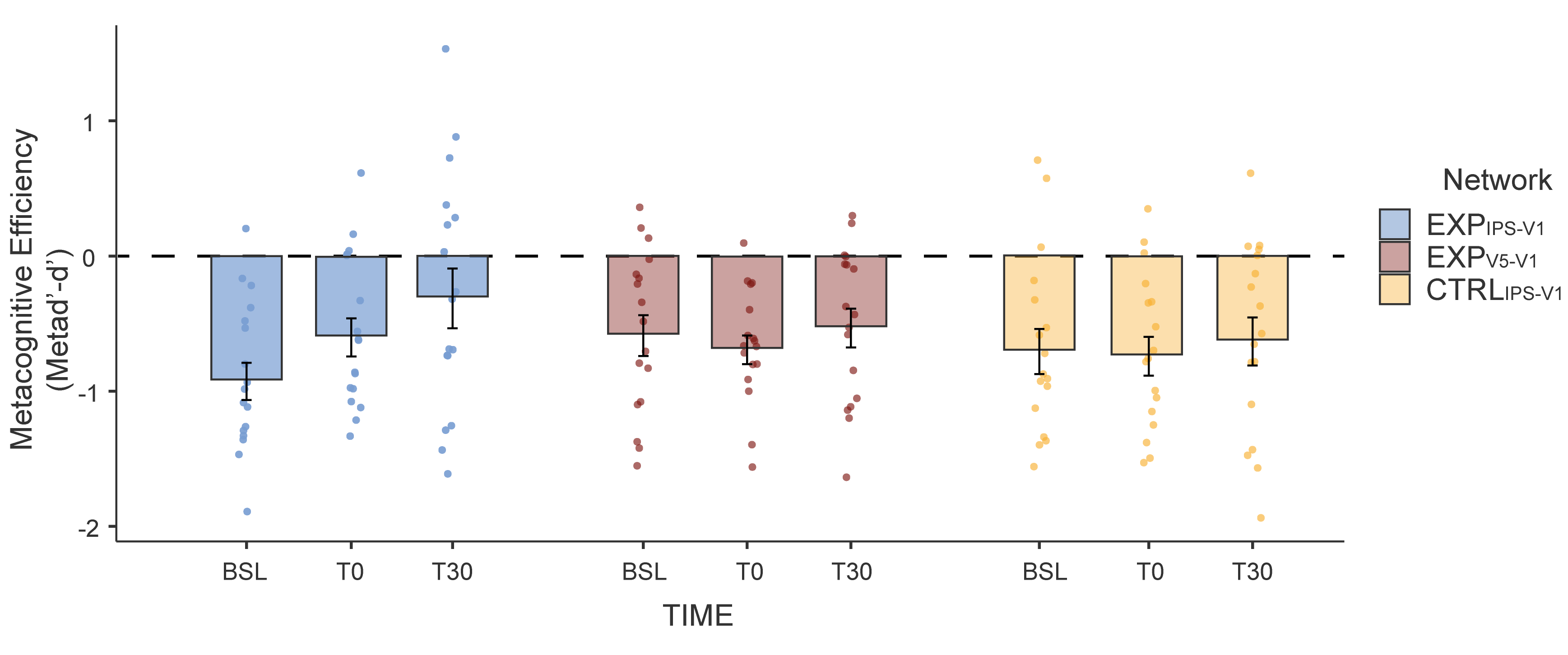

Supplement: S5 Fig — Negative values of efficiency represent suboptimal metacognition (meta-d’<d’), positive values indicate “hyper” metacognition (meta-d’>d’), whereas null values (meta-d’ = d’) means “ideal” metacognition. Filled bars and dots represent mean and individual subject performances, respectively. Data underlying this figure can be found in OSF: https://osf.io/x7d2e/?view_only=ac2ff19b1ab6415cb471895854fb5a35. (TIF) [file pbio.3001750.s005.tif]

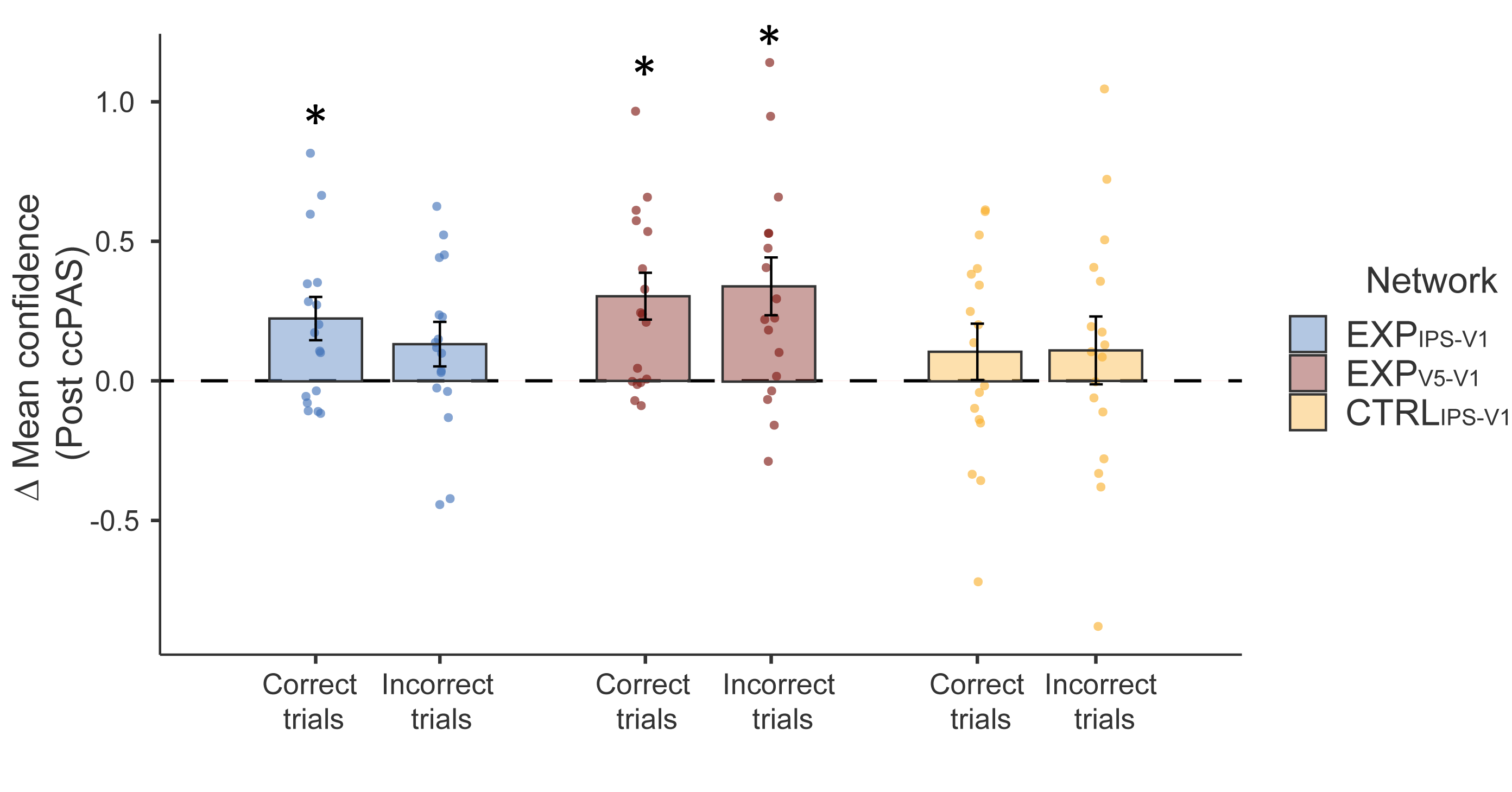

Supplement: S6 Fig — Average post-ccPAS confidence level as a function of correct (left side) and incorrect (right side) first-order performance. Filled bars represent mean (T0+T30) poststimulation values, and dots show individual subject performances. Asterisks point to significant p < .05 corrected one-sample t test. Data underlying this figure can be found in OSF: https://osf.io/x7d2e/?view_only=ac2ff19b1ab6415cb471895854fb5a35. (TIF) [file pbio.3001750.s006.tif]

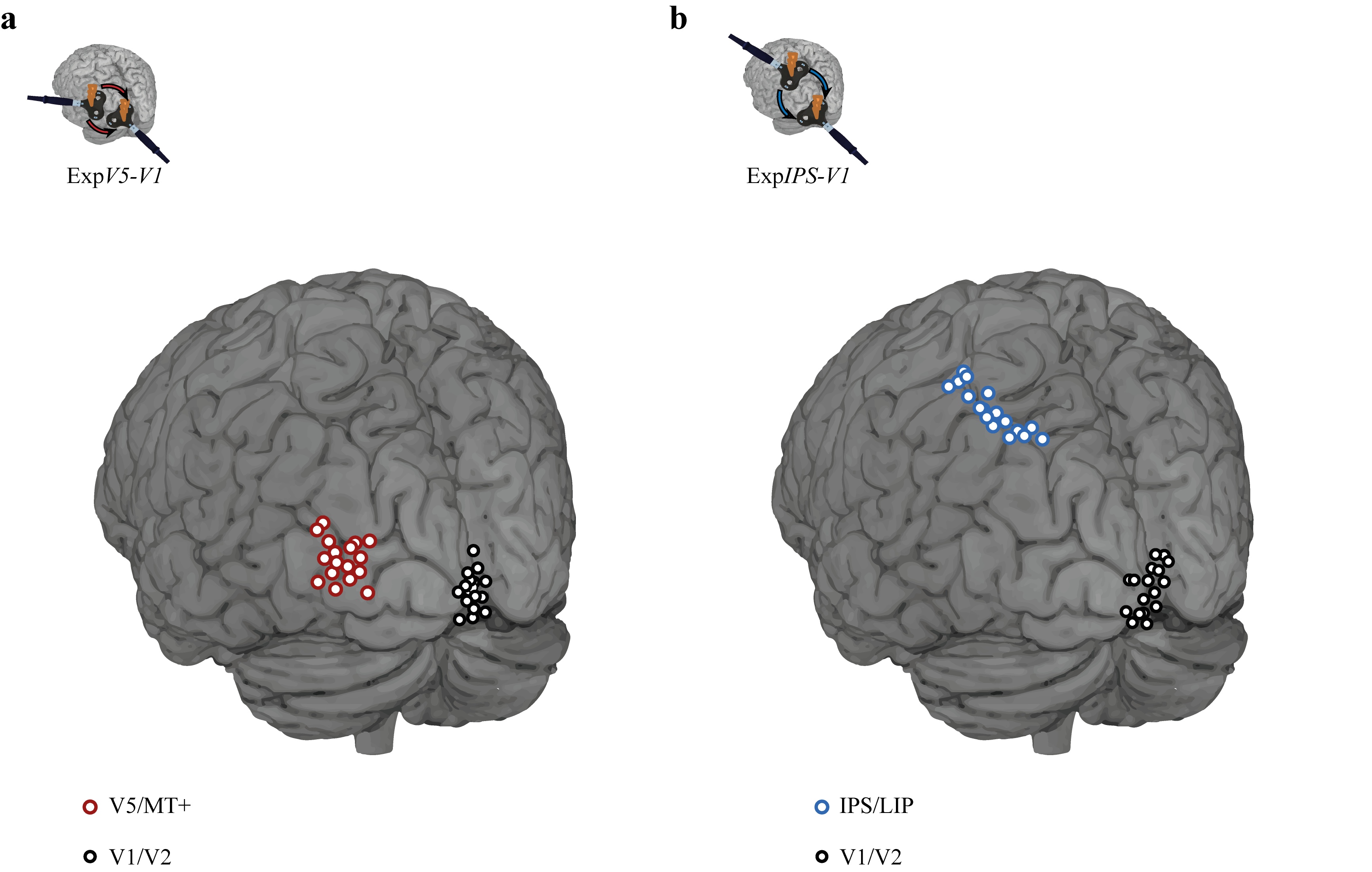

Supplement: S7 Fig — Single subjects’ coordinates of stimulation site for (a) V5/MT+ (red) and V1/V2 (black) and (b) IPS/LIP (blue) and V1/V2 (black), projected on a rendered brain surface from geometrical and EEG scalp positions reconstructed using MRICro (https://www.nitrc.org/projects/mricro). (TIF) [file pbio.3001750.s007.tif]
