# Supplementary material for: Human perceptual and metacognitive decision-making rely on distinct brain networks
Source: PLoS Biol. 2022 Aug 9;20(8):e3001750. doi: 10.1371/journal.pbio.3001750 (PMC9362930; doi:10.1371/journal.pbio.3001750)
Supplement: S1 File — Text A. Additional analysis on behavioral measure relative to first-order performance. Text B. Additional analysis on second-order performance. Text C. Supplementary discussion. Text D. Supplementary methods. Table A. Mean values ± SEM for hit rate (%), false alarm rate (%), criterion, and reaction times (ms) at each session, separately for condition. Table B. Mean R2 and slope ± SEM for each session, separately for condition. (DOCX) [file pbio.3001750.s008.docx]

**S1. Supporting results and methods.**

***Text A. Additional analysis on behavioral measure relative to first-order performance****.*

Complementary to the main relevant results regarding the motion psychometric threshold, we performed also additional analyses on overall detection rate (HR), false alarm rate (FAR), response times (RTs) and response criterion (c) to characterize first-order performance (see Table A for values across conditions). The ANOVA performed on baseline corrected HR values highlighted only a main effect of Targeted Network (F_2,48_ = 4.85; p = .012; np^2^ = .17), due to the increase of correct detection in Exp_V5-V1_ condition relative to Exp_IPS-V1_ (p = .005) and Ctrl_IPS-V1_ (p = .047) conditions. The ANOVA performed on baseline corrected FAR did not revealed any significant outcome attributable to the stimulation protocols (Fs < .67; ps > .51) but the main effect of the Session (F_1,48_ = 4.19; p = .046; np^2^ = .08), consisting in a negligible difference between T0 and T30. The ANOVA performed on baseline corrected ‘c’ did not showed any significant effect or interaction (Fs < 3.85; ps > .055). The ANOVA performed on RTs of first order choice did not show any significant effect or interaction (Fs < 1.72; ps > .19).

Finally, the ANOVA performed on the baseline corrected mean slopes of the psychometric curves (see Text D) did not return any significant effect (Fs < .87; ps >.42) (see Table B).

***Text B. Additional analysis on second-order performance.***

In order to better describe the nature of the metacognitive improvement observed, we included additional analyses on perceptual confidence. Specifically, we investigated confidence variations by taking into account the trial validity and by considering the correctness of response as a factor (estimating confidence ratings in error and correct trials). A clear discrepancy emerged when looking at the impact of different stimulation groups for correct and error responses in the post stimulus performance (avg T0+T30). Specifically, following IPS-to-V1 ccPAS, confidence increased exclusively for correct trials (t=2.87, p=.04), with no effect on incorrect trials (t=1.65, p=.12). Following V5-to-V1 ccPAS, confidence increased irrespective of correct (t=3.62, p=.01) and incorrect trials (t=3.29 p=.02).

To further shed light on the nature of the metacognitive modulation observed we have additionally tested metacognitive efficiency modulation was specific for near-threshold perceptual values or similarly also for above threshold values. The analysis includes the performance levels closest to the perceptual threshold (as for the main analysis) and the performance at higher contrast (i.e., asymptotic range from 35% to 80% coherence). Results of this analysis show a significant interaction Coherence*Network (F_2,48_=3.2; p=.049; np^2^ = .11) indicating that metacognitive modulations were dependent on the perceptual level. This resulted in a selective boost of metacognitive efficiency following IPS-to-V1 stimulation only at the critical level of performance (i.e., peri-threshold range) relative to matched peri-threshold levels of the Exp_V5-V1_ and Ctrl_IPS-V1_ conditions (ps < .048); on the other hand, we found an absence of modulation in conditions of higher discriminability (i.e., asymptotic range) between Exp_IPS-V1_ and the reciprocal asymptotic levels of Exp_V5-V1_ and Ctrl_IPS-V1_ (ps > .31).

Additionally, analysis performed on second-order RTs (i.e., following confidence rating) did not returned any significant effect nor interaction (Fs < .51; ps > .59).

***Text C. Supplementary Discussion***

These supplementary results suggest that an improved efficiency in metacognitive judgement found for IPS-to-V1 stimulation could be associated with a finer ability to correctly discriminate situations in which to assign high confidence (correct trial), from ambiguous situations where the sensory evidence available is not sufficient to provide adequate confidence (incorrect trial), an effect attributable to decreasing noise in confidence generation by the activity of gating on early visual cortex through parieto-occipital feedback pathways.

Regarding the outcome following V5-to-V1 stimulation, we argue that, following the increase of sensory signal for making a perceptual decision (enhancing motion sensitivity), the unspecific confidence gain might be induced by offsetting the overall impact of sensory noise leading to altered certainty readouts, with sensory information being overall more intense (hence, leading to higher confidence ratings). A potential explanation for these patterns of results may derive from probability distortions which lead to increasing over-or underestimation of certainty. Distortions are unlikely to cause variations in metacognitive efficiency because they are typically associated with systematic biases in confidence [1], in fact here it consistently led to overestimation.

***Text D. Supplementary Methods***

Additional behavioral measures of first-order choice were calculated by considering overall performance (excluding extreme levels of 0% and 80% coherence in order to avoid floor e ceiling effects), and were analyzed through ANOVAs on baseline-corrected values by considering the Targeted Network (Exp_V5-V1_, Exp_IPS-V1_ and Ctlr _IPS-V1_) and the Time from stimulation (T0, T30) as factors; Post-hoc analysis was performed using the Duncan test to correct for multiple comparisons. Psychometric slopes were calculated by taking the derivative of the logistic function [2] relative to the threshold level (75%) of performance, for each subject at each session. The slope of the psychometric function describes the steepness of the curve.

Perceptual confidence was computed by taking the average values across all ratings (i.e., 1-2-3-4), representing the overall tendency to be underconfident or overconfident. Analyses were conducted on baseline-corrected values and focused on performance level closest to the participants’ threshold, in order to characterize the main results of metacognitive efficiency. The mean confidence was evaluated first by considering all trials, and further splitting the trials according to their validity (correct vs incorrect).

**Supplementary Tables**

**Table A**

| **Condition** | **HR**  **(BSL)** | **HR**  **(T0)** | **HR**  **(T30)** |  | **FAR**  **(BSL)** | **FAR**  **(T0)** | **FAR**  **(T30)** |
| --- | --- | --- | --- | --- | --- | --- | --- |
| **Exp_V5-V1** | 83 ± 1 | 86 ± 1 | 86 ± 1 | **Exp_V5-V1** | 18 ± 2 | 16 ± 1 | 19 ± 2 |
| **Exp_IPS-V1** | 86 ± 1 | 85 ± 1 | 85 ± 1 | **Exp_IPS-V1** | 17 ± 2 | 18 ± 3 | 19 ± 2 |
| **Ctrl_IPS-V1** | 86 ± 1 | 86 ± 1 | 86 ± 1 | **Ctrl_IPS-V1** | 17 ± 2 | 15 ± 2 | 16 ± 2 |
| **Condition** | **C**  **(BSL)** | **C**  **(T0)** | **C**  **(T30)** |  | **RTs**  **(BSL)** | **RTs**  **(T0)** | **RTs**  **(T30)** |
| **Exp_V5-V1** | -.05 ± .07 | -.08 ± .06 | -.18 ± .06 | **Exp_V5-V1** | 547 ± .06 | 367 ± .04 | 349 ± .05 |
| **Exp_IPS-V1** | -.12 ± .08 | -.15 ± .09 | -.15 ± .06 | **Exp_IPS-V1** | 412 ± .05 | 321 ± .04 | 310 ± .05 |
| **Ctrl_IPS-V1** | -.08 ± .06 | .00 ± .07 | -.08 ± .07 | **Ctrl_IPS-V1** | 400 ± .05 | 273 ± .03 | 251 ± .03 |

Mean values ± s.e.m for hit rate (%), false alarm rate (%), criterion and reaction times (ms) at each session, separately for condition.

**Table B**

| **Condition** | **R^2^**  **(BSL)** | **R^2^**  **(T0)** | **R^2^**  **(T30)** |  | **Slope**  **(BSL)** | **Slope**  **(T0)** | **Slope**  **(T30)** |
| --- | --- | --- | --- | --- | --- | --- | --- |
| **Exp_V5-V1** | .94 ± .01 | .96 ± .01 | .95 ± .01 | **Exp_V5-V1** | .009 ± .001 | .011 ± .001 | .011 ± .001 |
| **Exp_IPS-V1** | .95 ± .01 | .95 ± .01 | .96 ± .01 | **Exp_IPS-V1** | .011 ± .002 | .011 ± .001 | .011 ± .001 |
| **Ctrl_IPS-V1** | .95 ± .01 | .94 ± .01 | .94 ± .01 | **Ctrl_IPS-V1** | .012 ± .001 | .011 ± .001 | .013 ± .001 |

Mean R^2^ and Slope ± s.e.m. for each session, separately for condition.

**Supplementary References**

1. Shekhar M, Rahnev D. The nature of metacognitive inefficiency in perceptual decision making. Psychol Rev. 2021;128: 45–70. doi:10.1037/REV0000249

2. Strasburger H. Converting between measures of slope of the psychometric function. Percept Psychophys 2001 638. 2001;63: 1348–1355. doi:10.3758/BF03194547
